# A thorough anion–π interaction study in biomolecules: on the importance of cooperativity effects†
†Electronic supplementary information (ESI) available: Fig. 1 and 2, Tables 1–24 and all interactions. See DOI: 10.1039/c5sc01386k


**DOI:** 10.1039/c5sc01386k

**Published:** 2015-06-05

**Authors:** Xavier Lucas, Antonio Bauzá, Antonio Frontera, David Quiñonero

**Affiliations:** a Pharmaceutical Bioinformatics , Institute of Pharmaceutical Sciences , Albert-Ludwigs-University , Hermann-Herder-Str. 9 , D-79104 Freiburg , Germany . Email: xavier.lucas@pharmazie.uni-freiburg.de ; Fax: +34 971173426 ; Tel: +34 971173498; b Departament de Química , Universitat de les Illes Balears , Crta. de Valldemossa km 7.5 , 07122 Palma de Mallorca , Spain . Email: david.quinonero@uib.es

## Abstract

Noncovalent interactions have a constitutive role in the science of intermolecular relationships, particularly those involving aromatic rings such as π–π and cation–π. In recent years, anion–π contact has also been recognized as a noncovalent bonding interaction with important implications in chemical processes. Yet, its involvement in biological processes has been scarcely reported. Herein we present a large-scale PDB analysis of the occurrence of anion–π interactions in proteins and nucleic acids. In addition we have gone a step further by considering the existence of cooperativity effects through the inclusion of a second noncovalent interaction, *i.e.* π-stacking, T-shaped, or cation–π interactions to form anion–π–π and anion–π–cation triads. The statistical analysis of the thousands of identified interactions reveals striking selectivities and subtle cooperativity effects among the anions, π-systems, and cations in a biological context. The reported results stress the importance of anion–π interactions and the cooperativity that arises from ternary contacts in key biological processes, such as protein folding and function and nucleic acids–protein and protein–protein recognition. We include examples of anion–π interactions and triads putatively involved in enzymatic catalysis, epigenetic gene regulation, antigen–antibody recognition, and protein dimerization.

## Abstract 2

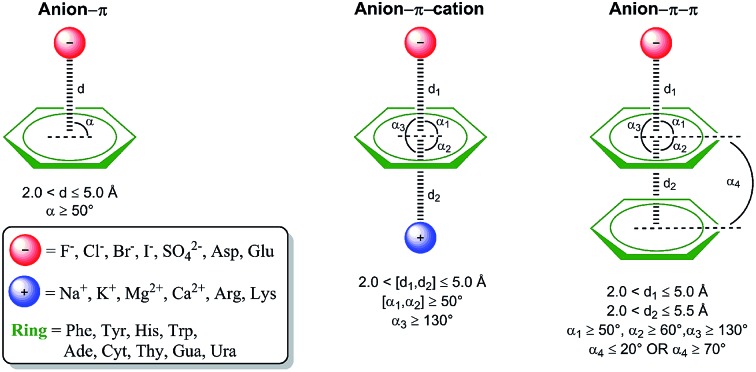
The importance of anion–π interactions in key biological processes is reported from a PDB analysis of anion–π interactions in biomolecules, also considering cooperativity effects by including other interactions.

## 1 Introduction

Noncovalent interactions have a constitutive role in the science of intermolecular relationships. In particular, those involving aromatic rings play a vital role in chemistry and biology,^1^ which becomes prominent in drug–receptor interactions, crystal engineering, and protein folding.^2^ For example, we have recently reported the small molecule XD14, a BET bromodomain inhibitor, which presents a key T-shaped π–π interaction with a tryptophan in the recognition site of the target responsible for high potency and selectivity.^3^ Around 60% of aromatic amino acid side chains (phenylalanine, tyrosine, tryptophan, and histidine) are estimated to participate in π-stacking interactions in proteins.^4^ Stacking interactions in nucleic acids play a fundamental role, wherein the structure of DNA duplexes is stabilized by nucleobase intra- and inter-strand stacking interactions.^4^,^5^ Moreover, the action of intercalating drugs as well as the biochemical processes implicated in the control and regulation of gene expression depend on protein–DNA stacking interactions.^6^ An additional related function takes place at the active site of a number of DNA repair enzymes, where alkylated purines are excised by means of a recognition mechanism based on π–π contacts with the side chains of aromatic amino acids.^7^ Similarly, these contacts play a crucial role in the repair process, where the insertion of aromatic amino acids into the DNA strand help preserve stability when the damaged base is flipped into the active site of the repair enzyme and out of the duplex.^7^

In recent years, the interaction between an electron-deficient aromatic moiety and an anion conveniently located above the ring plane has been accepted as a noncovalent bonding contact. The nature of this contact, designated an “anion–π interaction”,^8^ has been reported by a myriad of computational investigations, that prove that it is energetically favorable,^8^–^13^ as well as several experimental studies.^14^–^17^

Though the role of anion–π interactions in chemical processes is being progressively acknowledged,^18^–^23^ their involvement in biological processes has been scarcely reported. The search for anion–π interactions in biological macromolecules began in 2011, when our group reported clear evidence of anion–π interactions in the active site of urate oxidase, causing inhibition of the enzymatic activity, and thereby demonstrating the crucial role of this noncovalent interaction in a biological system for the first time.^24^ Three additional studies appeared the same year indicating that such interactions may be important in protein structures. A pioneering systematic search through the Protein Data Bank (PDB) showed that anion–π close contacts exist in experimental protein structures between the standard aromatic residues (Trp, Phe, Tyr, and His) and anions, such as chloride and phosphate.^25^ Also, by a systematic search of protein structures followed by *ab initio* calculations, our group showed that anion–π interactions are likely to occur in flavin-dependent enzymes.^26^ By examining high-resolution structures of proteins and nucleic acids, Chakravarty and co-workers pointed out that “η^6^”-type anion–π interaction is observed unambiguously and suggested it plays an important role in macromolecular folding and function.^27^ Howell and co-workers also performed a PDB search focusing on interactions between Phe and negatively charged residues such as Asp and Glu, leading to the conclusion that anion–π interactions are weakly attractive or slightly repulsive.^28^ A subsequent refinement of their PDB study for anion–π interactions showed that these interactions are present in thousands of protein structures with strong binding energies, as large as –8.7 kcal mol^–1^.^29^ Wetmore and co-workers thoroughly studied the interaction between cytosine and Asp or Glu concluding that the large magnitude of the anion–π interaction, up to *ca.* 23 kcal mol^–1^, suggests that it can play a large role in biology.^30^ Our group, on the one hand, also reported the critical role of the anion–π interaction in the mechanism of sulfide:quinone oxidoreductase,^31^ and, on the other hand, we demonstrated the importance of the anion–π interaction in the mechanism of inhibition of phenyldiketo acids of malate synthase.^32^

To dig deep into the current knowledge and understanding of the biological role of the anion–π interaction and greatly expand the number of possible interactions by increasing the number of interacting units, in this work we present a large-scale PDB analysis of the occurrence of anion–π interactions in proteins and nucleic acids, by considering the side chains of Phe, Tyr, Trp, and His and the purine and pyrimidine bases as the interacting aromatic rings, and F^–^, Cl^–^, Br^–^, I^–^, SO_4_^2–^, PO_4_^3–^, NO_3_^–^, CO_3_^2–^, Glu, and Asp as the interacting anions (because the p*K*_a_ values for Asp and Glu are low, 3.5–4.5,^33^ we assume Asp and Glu are always ionized). Moreover, to gain insight into the role of anion–π interactions in the stabilization of macromolecular complexes, inter-chain recognition has also been a subject of study, primarily for proteins. We have gone a step further in the analysis by considering the existence of cooperativity effects through the inclusion of a second noncovalent interaction, *i.e.* π-stacking, T-shaped, or cation–π interactions. These cooperativity effects are supposed to be of utmost importance for the weakly attractive anion–π interactions where the aromatic ring is an electron-rich π-system. As far as we are aware this is the first time that cooperativity effects are addressed in a study of anion–π interactions in biological systems.

## 2 Results and discussion

**Fig. 1:**
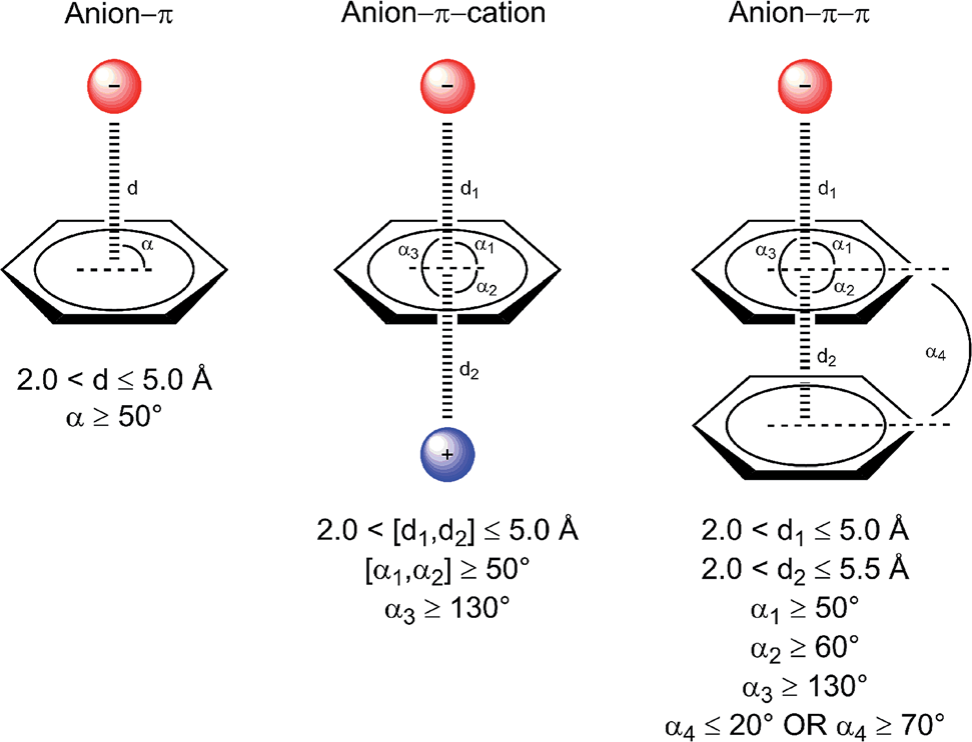
Considered interaction types and geometric parameters used during data collection. Distances *d*_a_, *d*_c_, and *d*_p_ are between the centroid of the aromatic ring and the anion, cation, and centroid of another aromatic ring, respectively. Angles *α*_a_, *α*_c_, and *α*_p_, are formed by the vector connecting the ring centroid with the anion, cation, and another ring plane, respectively. Angle *α*_pp_, is formed between ring planes. A comprehensive definition of centers and centroids for each amino acid, nucleic base, and ion is offered as ESI Table 1.†

Geometric parameters used during data collection are depicted in Fig. 1, along with exemplary binary and ternary interactions. The search yielded thousands of anion–π interactions contained in the PDB, as well as ternary complexes involving additional aromatic systems and cations. The identified interactions are summarized in Table 1 and the complete lists are offered as ESI.†

**Table 1:** Considered interaction types and their frequency in the processed PDB structures

Interaction type	Involving DNA	Involving RNA	Involving only proteins	Involving different protein chains
Anion–π	69	197	82 456	5395
Anion–π–cation	0	15	2398	264
Anion–π–π	59	21	2945	354

### 2.1. Binary anion–π interactions

#### 2.1.1. Interactions involving DNA

In ESI Tables 2 and 3† we include the interacting residues and summarize the results of the search of anion–π interactions with adenine (DA), cytosine (DC), thymine (DT), and guanine (DG) rings as in DNA. First, we observed 69 interactions in 56 unique PDB structures, 63 of which corresponded to protein–DNA complexes. We could not detect selectivity towards either of the two most abundant anions, *i.e.* Glu (32 interactions) and Asp (31 interactions), accounting for 91.3% of interactions. For the rest of the anions, namely, chloride and sulfate, only 1 and 5 anion–π interactions were found, respectively. The most representative binary contact is Glu–DT, followed by Asp–DC and Asp–DT. From the results in ESI Table 3† it can be extracted that the interactions with DT and DC are, by far, the most numerous: 84.3% and 96.8% of Glu and Asp, respectively, interact *via* anion–π contacts with the pyrimidinic rings. The purine bases adenine (6 hits) and guanine (1 hit) barely establish interactions. From the electrostatic point of view, it is understandable why the most π-acidic ring, thymine, is the most abundant interacting residue. However, the electrostatic contribution alone cannot explain the disparity of cytosine and guanine rings.

**Fig. 2:**
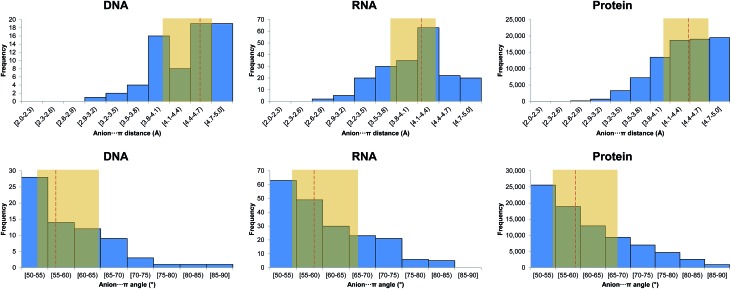
Histograms of equilibrium distance (top) and angle (bottom) for DNA, RNA, and protein binary anion–π interactions. Position of the median is shown as a dashed red line, and the interquartile range is depicted as a shadowed yellow area.

In Fig. 2 we show histograms of the equilibrium distance and angle as defined in the computational methods. Overall, we observe that the median equilibrium distance (*d*^–^) and angle (*ᾱ*^–^) are 4.53 Å and 56.2°. If we break down these values into the different anion contributions, the shortest *d*^–^ and smallest *ᾱ*^–^ are found for Glu with 4.39 Å and 61.6°. Intriguingly, the interactions with Asp have a sensibly longer *d*^–^ (4.67 Å) along with a wider *ᾱ*^–^ (53.4°) than Glu, which can only be attributed to the longer Glu side chain. Sulfate presents a *d*^–^ of 4.50 Å and the largest *ᾱ*^–^, 55.1°.

We have also analyzed the orientation of the carboxylate with respect to the aromatic system by considering the angle between the plane defined by the carboxylic carbon and oxygen atoms in Asp and Glu and the plane of the interacting aromatic system. The results are gathered in ESI Fig. 2.† A value close to 0° indicates a face-to-face interaction and a value close to 90° indicates an edge-to-face interaction. As inferred from the inspection of the figure a face-to-face approach predominates with a value for the median angle of 30.0°, which is consistent with a reinforcement of the anion–π interaction by a π–π effect.

**Fig. 3:**
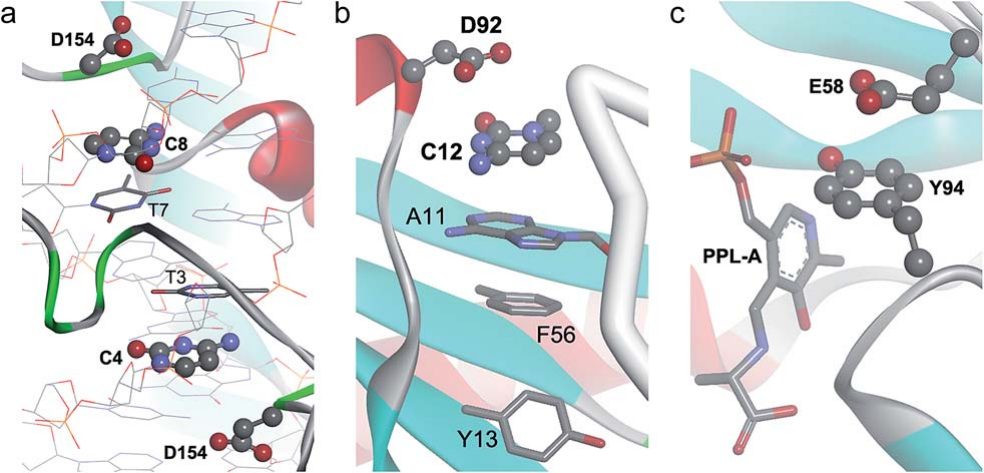
Binary anion–π interactions in biological systems. (a) Post-reactive state of endonuclease *Bam*HI complexed to DNA (PDB code ; 3BAM), where Asp–π interactions are shown. (b) C-terminal domain of human protein U1A bound to RNA (PDB code ; 1URN), where the Asp92–π interaction is shown. (c) Active site of the complex of pyridoxal-5′-phosphate-dependent catalytic antibody ; 15A9 with a phosphopyridoxyl-l-alanine (PPL-A) substrate analogue (PDB code ; 1WC7), where the Glu58–π interaction is shown.

An example of anion–π interaction is illustrated in Fig. 3a, with the *Bam*HI type II restriction endonuclease bound to DNA in the presence of Mn^2+^ and Ca^2+^.^34^ Type II restriction endonucleases are phosphodiesterases that recognize short palindromic DNA sequences and cleave both DNA strands to yield 5′-phosphate and 3′-hydroxyl groups. In the figure, two anion–π interactions between Asp154 and cytosines 4 and 8 from different strands are shown for the post-reactive state of the enzyme. The pre-reactive state of the enzyme preserves the same two interactions,^34^ which also appear in a previous structure of *Bam*HI in the absence of metals.^35^ An overlay of the enzyme in its *apo* form and in the pre-reactive state reveals that, upon DNA binding, Asp154 is displaced by 5.77 Å to engage in an anion–π interaction with cytosine (ESI Fig. 1†).

#### 2.1.2. Interactions involving RNA

The results obtained from the search of anion–π interactions with adenine (A), cytosine (C), uracil (U), and guanine (G) rings as in RNA are gathered in ESI Tables 4 and 5.† We observed 197 interactions in 69 unique PDB structures, 177 of which corresponded to protein–RNA complexes. Glu (123 interactions, 62.4%) selectively interacts *via* anion–π over Asp (the second most abundant anion, 55 interactions, 27.9%), with both anions accounting for 90.3% of interactions. These results are in striking contrast with DNA results, where no selectivity for either Glu or Asp was observed (ESI Table 3†). Apart from Glu and Asp, the other anions that appear from the search are chloride and sulfate, with 13 and 6 anion–π interactions, respectively. As opposed to the DNA results, the most common contact pair is Glu–A, which represents 32.5% of all the contact pairs.

From the results gathered in ESI Table 4† it can be deduced that A (92.8%), G (65.2%), and U (71.1%) interact preferentially with Glu. If we only consider surface (inter-chain) interactions by removing Cl^–^ and SO_4_^2–^, all these percentages are moderately increased except for guanine which dramatically increases up to 96.8%. The statistical analysis unveils an unexpected preference of cytosine to interact with Asp: 90.9% of cytosine forms anion–π interactions with the amino acid. This preference is reciprocal because 72.7% of Asp is found in anion–π contacts with cytosine. These results are also supported by the expected small numbers of Glu–A and Asp–C pairs. This enrichment induces a significant reduction of pairs of Asp with the purine bases adenine and guanine, yet it does not affect the formation of complexes with uracil. Chloride shows the highest selectivity with all 13 anions interacting with the guanine ring, as can be also inferred from the comparison of the expected and actual amounts for the Cl–G pair.

All these results are in stark contrast with the DNA results since in RNA there is not a predominance of pyrimidine over purine bases. These impaired selectivities can only be justified by differences between the nucleic acids. On the one hand, they might depend on the conformational effect derived from C3′-endo (DNA) or C2′-endo (RNA) sugar puckering that leads to different distances and twist angles between two subsequent base pairs along the helical axis. On the other hand, it needs to be born in mind that the unbalanced amount of anion–π interactions identified in RNA and DNA within the PDB (Table 1) may lead to biased conclusions.

The histograms of the equilibrium distance and angle are shown in Fig. 2. First, we observe that the distribution of the interaction distances in RNA and DNA is remarkably distinct. Indeed, *d*^–^ is much shorter in RNA (Δ*d*^–^ = –0.39 Å). If we pay attention to the different anions separately, the shortest *d*^–^ with a small *ᾱ*^–^ is found for Asp, with 3.85 Å and 67.1°. In contrast to DNA, it is worth mentioning that the interactions with Glu have a much longer *d*^–^ (4.21 Å) along with a much wider *ᾱ*^–^ (55.5°) than Asp.

The orientation of Asp and Glu carboxylates with respect to the aromatic system (ESI Fig. 2†) shows that a face-to-face approach is dominant, with a value for the median angle of 20.3°. The angle is smaller than in DNA, indicating a strong reinforcement of the anion–π interaction when it interacts planar to RNA bases.

In Fig. 3b we show an example of an anion–π interaction for RNA–U1A human protein binding, which is critical in the transcription process of genetic information.^36^ Experimentally it is known that the C-terminal domain (which includes the Asp92 residue) is crucial for the stability of the RNA–U1A complex.^37^ It has been demonstrated^38^ that this binding mechanism is primarily based on an anion–π interaction between Asp92 and C12, which seems to be critical in controlling the locking/unlocking binding mechanism in the RNA-binding specificity of human U1A protein.

#### 2.1.3. Interactions involving proteins

The results obtained from the search of anion–π interactions with the side chains of histidine (His), phenylalanine (Phe), tyrosine (Tyr), and tryptophan (Trp) as found in proteins are summarized in Table 2 and ESI Tables 6 and 7.† We observed 82 456 interactions in 38 027 unique PDB structures, 80 346 of which corresponded to interactions exclusively involving amino acids. It is noteworthy that these results imply that 61.3% of all the processed structures in the PDB (62 033 structures, Methods) contain anion–π interactions as classified herein. The ratio of Glu compared to Asp in such interactions is slightly greater (46 132 interactions, 55.9%) than the total percentage of Glu in our working PDB set (51.7%, ESI Table 8†), indicating a modest selectivity for this anion to be entangled in anion–π interactions. These results are similar to those obtained for RNA, though the selectivity for Glu is higher in RNA than in proteins. In addition to Glu and Asp, the rest of the identified anions involved in the interactions include sulfate, chloride, phosphate, with 1055 (1.3%), 627 (0.8%), 261 (0.3%), respectively, and minute amounts of nitrate, carbonate, bromide, and fluoride. The relative amounts of sulfate, chloride, and phosphate anions interacting with π-systems are larger than the relative amounts of these anions in the PDB (0.9%, 0.4%, and 0.1%, respectively), indicating an enrichment of those anions in the π-interactions with proteins.

**Table 2:** The most common binary anion–π interactions in proteins. Pairs of interacting residues and their occurrences in number (amount), percentage (%), and residues' representativities for each distinct anion (%A^–^) and π-system (%π). The expected amount of each interaction pair, according to its relative abundance, and the statistical significance are shown (Methods). Statistical significance is denoted with ** for *p*-value < 0.01, and *** for *p*-value < 0.001

Interaction	Amount (*expected*)	%	%A^–^	%π
Glu–His	13 763 (13 805)	16.7	29.8	55.8
Glu–Tyr	13 592 (13 428)	16.5	29.5	56.6
Glu–Phe	13 060 (13 446)**	15.8	28.3	54.3
Asp–Phe	10 477 (9972)***	12.7	30.6	43.6
Asp–His	10 180 (10 239)	12.3	29.8	41.3
Asp–Tyr	9797 (9959)	11.9	28.6	40.6
Glu–Trp	5717 (5450)**	6.9	12.4	58.7
Asp–Trp	3760 (4042)**	4.6	11.0	38.6
SO_4_–His	405 (316)**	0.5	38.4	1.6
SO_4_–Tyr	274 (307)	0.3	26.0	1.1

The most abundant aromatic amino acid in the PDB is Phe (35.2%), followed by Tyr (31.0%), His (21.0%), and Trp (12.8%) (ESI Table 8†). For His and Phe this distribution varies when only those amino acid side chains that are involved in anion–π interactions are taken into account (ESI Table 6†): His is the most abundant residue, which appears in 29.9% of the cases, in detriment of Phe (29.1%). This is consistent with the existence of protonated imidazole moieties at physiological pH thus favoring the electrostatic contribution of the anion–π interaction.

There is no contact pair that stands out from the rest (Table 2), in contrast to what is observed in DNA and RNA (ESI Tables 3 and 4†): Glu–His, Glu–Tyr, and Glu–Phe are the most numerous pairs, approximately contributing 16% each. The corresponding pairs of Asp with His, Tyr, and Phe account for around 12% each. From the inspection of the results in Table 2, it can be reasoned that Glu and Asp have the same preference for the aromatic rings of Phe, His, Tyr, and Trp for establishing anion–π interactions. For example, 29.8% of both Glu and Asp establish anion–π interactions with His. Almost identical percentages are obtained for Phe and Tyr, regardless of whether the anion is Glu or Asp. However, a closer look at the absolute values reveals subtle differences: Phe preferably attracts Asp instead of Glu, *i.e.* the abundance of Glu–Phe interactions is significantly lower than expected, which is compensated by a higher occurrence of Asp–Phe pairs. Conversely, Trp presents a tendency to interact with Glu instead of Asp.

**Fig. 4:**
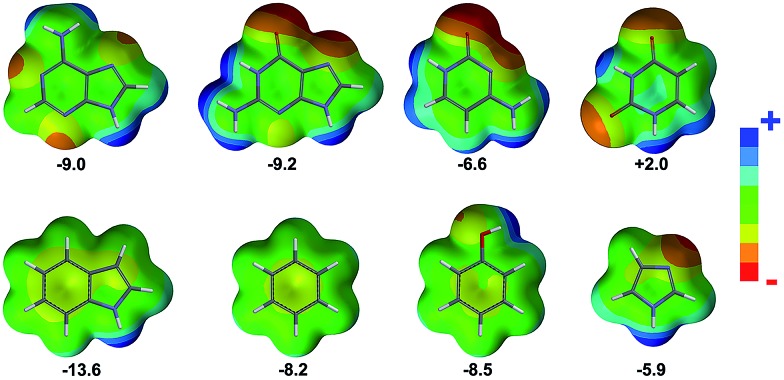
Molecular electrostatic potential surfaces. From left to right and from top to bottom: adenine, guanine, cytosine, uracil, indole, benzene, phenol, and imidazole, as simplified representations of nucleotides, deoxynucleotides, and side chains of the aromatic amino acids studied herein. The value of the component of the quadrupole moment (in Buckinghams) perpendicular to the ring is shown.

In Fig. 2 the histograms of the equilibrium distance and angle are shown. First, it is worth noting the larger *d*^–^ in proteins compared to RNA (4.36 Å and 4.14 Å, respectively). To some extent this difference could be anticipated because the nucleobases are more π-acidic than the phenyl, imidazole, and indole rings, as can be inferred from the electrostatic potential surface maps shown in Fig. 4. However, *d*^–^ for DNA is a little bit longer than for proteins (Δ*d*^–^ = 0.17 Å), which may be due to a bias resulting from the small amount of DNA data (69 interactions). If we partition the results in terms of anion contributions, both Glu and Asp have very similar *d*^–^ values (4.38 Å and 4.33 Å, respectively), whereas nitrate, carbonate, and phosphate exhibit the shortest *d*^–^ (3.93 Å, 3.96 Å, and 4.04 Å, respectively). In addition, if we analyze the results in terms of amino acid contributions, the shortest *d*^–^ is found for His (4.16 Å), as expected from electrostatic considerations (Fig. 4), whereas very similar yet longer distances are found for Phe, Trp, and Tyr (4.41 Å, 4.40 Å, and 4.44 Å, respectively). The strong electrostatic interactions of His are analogously directing its engagement with sulfate and phosphate: SO_4_/PO_4_–His pairs are favored compared to SO_4_/PO_4_–Tyr and SO_4_/PO_4_–Phe.

Analogously to DNA and RNA, the orientation of Asp and Glu carboxylates with respect to the interacting aromatic system (ESI Fig. 2†) reveals a clearly dominant face-to-face approach, with a value for the median angle of 31.1°.

In Fig. 3c a partial view of one of the two active sites of the complex of the pyridoxal-5′-phosphate (PLP)-dependent catalytic antibody ; 15A9 with a phosphopyridoxyl-l-alanine (PPL-l-Ala) substrate analogue is illustrated.^39^ The antibody catalyzes, in addition to Schiff base formation, transamination, and α-, β-elimination reactions.^40^,^41^ As shown in the figure, Tyr94 is interacting both with the substrate *via* hydrogen bonding and Glu58 *via* anion–π contact.

#### 2.1.4. Interactions involving protein surfaces

The results when only inter-chain anion–π interactions are considered, either as part of the same protein or in protein–protein complexes, are summarized in ESI Tables 9 and 10.† We retrieved 5395 surface interactions of a total of 82 456 interactions. Therefore a remarkable 6.5% of all the anion–π interactions in proteins are established between amino acids of different chains of one or more proteins, leading to the conclusion that anion–π contacts play an active role in protein interface recognition and have an underestimated contribution in protein–protein interactions. The percentage of Glu is similar, though slightly greater, than that observed in the general protein search, with Glu being the major anion (60.3%). The abundance of the four aromatic amino acids varies with respect to those obtained from the general search, along with their relative ordering. In chain interfaces the most abundant amino acid is Tyr (His in the general search), appearing in 35.0% of the cases, followed by Phe (28.7%), His (28.5%), and Trp (7.8%). As a consequence of these results, the most common amino acid pair is Glu–Tyr with an occurrence of 21.5% (ESI Table 9†).

The analysis of the geometrical parameters for the inter-chain anion–π interactions yields results similar to those obtained for the general protein search.

### 2.2. Ternary anion–π interactions in DNA

#### 2.2.1. Anion–π–cation

The analysis of the anion–π–cation interactions in DNA could not be performed because the search returned no successful hits (Table 1).

#### 2.2.2. Anion–π–π

The search for anion–π–π interactions in DNA returned 59 successful hits, 38 of which are the result of binary anion–π interactions forming triads with an additional DNA base (ESI Table 11†). Therefore 55.1% of the anion–π interacting aromatic systems from the parent binary anion–π interaction are further involved in π–π interactions with DNA. The remaining 21 hits are of the anion–π(protein)–π(DNA) type. It is worth mentioning that all 59 aromatic interactions are of the π-stacking type. In terms of anion and nucleic base representativities, the results are similar to those obtained for the parent anion–π search (ESI Tables 3 and 11†). However, the partition of the π-donor systems into those that are central (π_c_) and terminal (π_t_) gives insights into the specific attraction of the aromatic groups for the central and terminal positions of the ternary anion–π–π complexes and their combined cooperativity effects: adenine (1 hit) and guanine (0 hits) barely establish anion–π interactions, yet they are attracted to anion–π interactions to form ternary complexes (19 and 12 hits, respectively). Conversely, cytosin (1 hit) and His (0 hits) barely establish π–π interactions, and rather participate in ternary complexes with DNA occupying the central location (18 hits each). Intriguingly, His only interacts with Asp to form triads with DNA, despite the higher amount of Glu–His anion–π interactions in proteins compared to Asp–His (Table 2). However, the purine bases are important contributors to the π–π interactions. In fact, adenine, guanine, and especially thymine represent, respectively, 32.2%, 20.3%, and 44.1% of all the aromatic rings entangled in π–π interactions (ESI Table 12†). The study of geometrical parameters for the anion–π and π–π interactions revealed a *d*^–^ (4.61 Å) similar to that of the corresponding binary interaction, and a median π–π equilibrium distance (*d*^π–π^) of 3.60 Å.

A representative example of an anion–π–π interaction is illustrated in Fig. 3a, where the *Bam*HI type II restriction endonuclease is shown bound to DNA.^34^ In the figure, in addition to the two anion–π interactions with cytosine described above (section 2.1.1), we observe how these π-systems simultaneously establish π–π interactions with thymine.

### 2.3. Ternary anion–π interactions in RNA

#### 2.3.1. Anion–π–cation

The search for anion–π–cation interactions in RNA returned 15 successful hits out of 197 anion–π interactions (ESI Tables 13 and 14†), *i.e.* 7.6% of the aromatic systems are involved in additional cation–π interactions.

The results show a *d*^–^ (3.57 Å) considerably shorter than that reported for the parent Glu–π binary interaction (4.21 Å). This result suggests that the anion–π interaction is substantially strengthened when the π-system additionally interacts with a cation on the opposite side of the ring, leading to a cooperative effect. Previous studies have shown similar cooperativity effects in systems where either benzene or hexafluorobenzene simultaneously interacts with an anion on one side of the ring plane and a cation on the opposite side:^42^,^43^ in the present study the median cation–π distance (*d*^+^) is 3.77 Å, which could be considered quite long. However, it has to be borne in mind that herein this geometrical parameter is not defined as the minimum distance between the cation and the ring plane (ESI Table 1†). Moreover, the median cation–π angle (*ᾱ*^+^ = 72.8°) is larger than the corresponding angle for the anion–π interaction (*ᾱ*^–^ = 59.5°). This is in agreement with the different directionality of both interactions: in anion–π complexes the displacement of the anion along the parallel plane does not imply such a large interaction energy loss (≤7%) compared to the cation–π complexes (≤23%).^44^

**Fig. 5:**
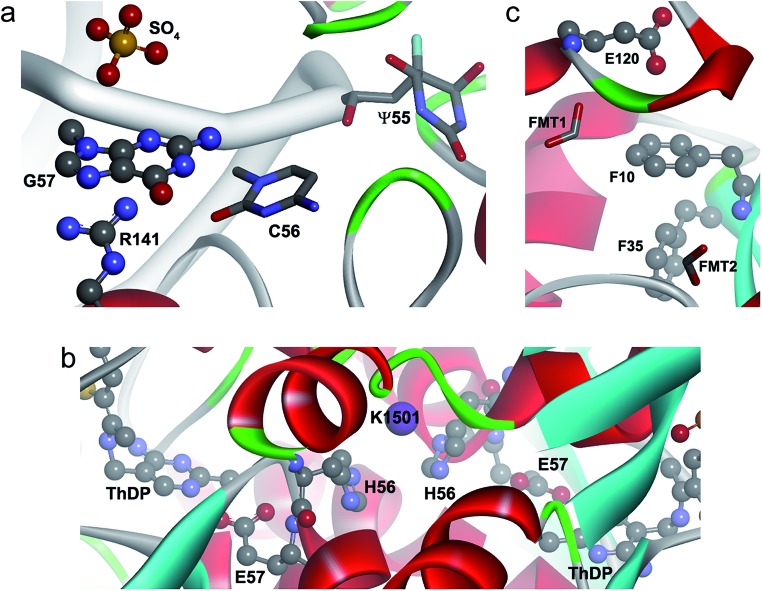
Ternary anion–π–cation and anion–π–π interactions in biological systems. (a) Pseudouridine synthase TruB complexed to RNA (PDB code 1K8W), where SO_4_–π–Arg interactions are shown. (b) Active site of CEAS complexed to dipotassium l-(+)-tartrate (PDB code ; 2IHU), where Glu–π–K^+^ interactions are shown. (c) Active site of PfGST (PDB code ; 1OKT), where a Glu–π–π interaction is shown.

A representative example of an anion–π–cation interaction is illustrated in Fig. 5a.^45^ Pseudouridine (Ψ) synthases catalyze the isomerization of specific uridines in cellular RNAs to pseudouridines and may function as RNA chaperones. The TruB cocrystal structure reveals that this Ψ synthase gains access to its substrate by flipping out nucleotide 55 of tRNA. In addition TruB binding flips out two additional nucleotides, namely C56 and G57, which may keep the ribose of U55 from flipping back prematurely before reattachment to the rotated nucleobase. Within this context, the anion–π interaction depicted in the figure is formed between one of the multiple sulfate anions that appear in the crystal and the flipped-out G57 which, at the same time, is cation–π interacting with the guanidinium side chain of Arg141.

#### 2.3.2. Anion–π–π

The search for anion–π–π interactions in RNA returned 26 hits out of 197 anion–π interactions, *i.e.* 13.2% of the anion–π interacting aromatic systems are involved in π–π interactions (ESI Tables 15 and 16†). All identified interactions are of the π-stacking type. As in DNA, chloride is missing in the ternary results. The relative amounts of the anions show significant changes with respect to the parent anion–π interaction results (ESI Table 4†), where Glu was the major anion: There is a high selectivity towards Asp (65% of the interactions). The relative weight of π_c_ is also quite different from that found for the parent binary interactions, now yielding C as the major contributor (14 interactions), followed by U (4 interactions), and G (2 interactions). Intriguingly A is rarely observed in the central location of ternary complexes (1 interaction) despite its high abundance in the binary systems. Conversely, adenine is the most common π_t_, with 13 interactions, indicating its affinity to form ternary complexes with already established anion–π systems. Consequently, Asp–C–A is the most abundant ternary contact, representing almost 50% of all triads. In addition, and similar to the parent binary interaction, 76.5% of Asp is anion–π interacting with C. U establishes 6 π–π interactions as a terminal moiety, mainly with His in the Glu–His–U triad.

In Fig. 3b we show a snapshot of RNA recognition by U1A human protein.^36^ As previously described, the binding mechanism is primarily based on an anion–π interaction between Asp92 and C12. Additionally, the cytosine is π–π interacting with an adenine π-system (A11), suggesting electronic cooperativity effects in the locking/unlocking RNA-binding mechanism.

### 2.4. Ternary anion–π interactions in proteins

#### 2.4.1. Anion–π–cation

The search for anion–π–cation interactions in proteins returned 2398 hits out of 82 456 anion–π interactions, implying that 2.9% of the aromatic systems are involved in cation–π interactions (Table 3 and ESI Table 18†). The anions that participate in the interactions are Glu, Asp, sulfate, chloride, phosphate, and nitrate. Among them, Glu is the most numerous (49.3%, ESI Table 17†) followed by Asp (47.8%). This contrasts with the parent binary interaction, where Glu is more abundant (ESI Table 6†). However, the distribution of π_c_ is similar to that found in the binary anion–π interactions.

**Table 3:** The most common ternary anion–π–cation interactions in proteins. Triads of interacting residues and their occurrences in number (amount), percentage (%), and residues' representativities for each distinct anion (%A^–^), central π-system (%π_c_), and cation (%C^+^). The expected amount of each interaction pair, according to its relative abundance, and the statistical significance are shown (Methods). Statistical significance is denoted with ** for *p*-value < 0.01, and *** for *p*-value < 0.001

Interaction	Amount (*expected*)	%	%A^–^	%π_c_	%C^+^
Asp–His–Arg	272 (211)**	11.4	23.7	42.6	16.5
Asp–Tyr–Arg	268 (240)	11.2	23.4	36.8	16.2
Glu–Tyr–Arg	250 (248)	10.4	21.1	34.3	15.1
Glu–Phe–Arg	244 (208)	10.2	20.6	39.9	14.8
Glu–His–Arg	189 (217)	7.9	16.0	29.6	11.4
Glu–Trp–Arg	175 (142)	7.3	14.8	41.8	10.6
Asp–Phe–Arg	130 (201)***	5.4	11.3	21.3	7.9
Asp–Phe–Lys	129 (86)**	5.4	11.2	21.1	18.2
Glu–Trp–Lys	115 (61)***	4.8	9.7	27.4	16.3
Asp–Tyr–Lys	106 (103)	4.4	9.2	14.5	15.0

As in RNA, Arg is the most abundant cation (68.9%) followed by Lys (29.5%). This is an interesting result because the total amount of Lys (1 816 877) in the PDB (ESI Table 8†) is slightly larger than that of Arg (1 631 104). Therefore the central aromatic system shows a clear preference for guanidinium rather than ammonium moieties. Similarly, several cation–π studies by Gromiha and coworkers show that Arg has a higher preference to form cation–π interactions than Lys and that the roles of these cation–π interactions are different from other noncovalent contacts in the stability of protein structures.^46^–^48^

The presence of Na^+^ and K^+^ is scarce, with only 35 and 4 interactions, respectively. The π–Na^+^ interactions appear in the Asp–Phe–Na triad (ESI Table 18†) and were retrieved from X-ray diffraction studies of β-galactosidase from *E. coli*. This enzyme catalyzes hydrolytic and transgalactosidic reactions on β-d-galactopyranosides. Likewise, the four K^+^ contacts are found in *N*^2^-(2-carboxyethyl)arginine synthase (CEAS), an unusual thiamin diphosphate (ThDP)-dependent enzyme that catalyzes the committed step in the biosynthesis of the β-lactamase inhibitor clavulanic acid in *Streptomyces clavuligerus*.^49^ Reaction mechanisms proposed for CEAS^50^–^52^ imply a ThDP-mediated catalysis where Glu57 is actively involved as a proton donor–acceptor. In the complex formed with the substrate analog dipotassium l-(+)-tartrate (Fig. 5b), Glu57 is anion–π interacting with His56, which in turn is cation–π interacting with K1501. This cooperatively-strengthened anion–π interaction, which went unnoticed by the authors, might be relevant to arrange Glu57 towards ThDP. Remarkably, K^+^ is perfectly accommodated between two His of different chains, His56C/His56D and His56A/His56B, in a space that is occupied by water molecules in the native state of the enzyme.

The results of all ternary contacts (Table 3 and ESI Table 18†) reveal four predominant triads (with abundances ranging from 10.2% to 11.4%), all comprising arginine as cation (Asp–His–Arg, Asp–Tyr–Arg, Glu–Tyr–Arg, and Glu–Phe–Arg). The rest of the contacts represent less than 8% each. If we compare these results with those of the binary interaction (Table 2), we observe that the relative weight of each anion–π contact has changed: the percentage of the interaction of Glu with Trp is 12% bigger in the ternary search, in detriment of the rest of the amino acids. Conversely, the percentage of the interaction of Asp with Tyr and His has been increased in detriment of the interaction with Phe and Trp. Regarding the π–cation contact pairs, Tyr–Arg alone represents 21.6% of all pairs, followed by His–Arg (19.3%), Phe–Arg (15.6%), and Trp–Arg (10.7%).

The *d*^–^ value is 4.38 Å, which is very similar to the reported value for the parent binary interaction. The *d*^+^ value (3.92 Å) is shorter than the related *d*^–^, consistent with the smaller radius of cations compared to anions. As expected, *ᾱ*^+^ (68.3°) is larger than the corresponding *ᾱ*^–^ (59.5°), as previously observed in RNA, which is in agreement with the different directionality of both interactions.^44^

The comparison and statistical analysis of the collected and expected amounts of each triad, based on the relative abundance of each interaction partner within the data set, provide interesting insights into otherwise hidden details on cooperativity effects for the ternary anion–π–cation gathered in Table 3. First, the most common ternary complex, Asp–His–Arg, is significantly enriched in detriment of the related Glu–His–(Arg/Lys), indicating again a higher preference of the Asp–His complex to form triads despite its lower abundance in the parent binary interaction (Table 2). Geometric data supports a strong synergistic effect for Asp–His–(Arg/Lys) compared to the Glu parent ternary complexes: the *d*^+^ value is substantially increased to 3.83 Å (Δ*d*^+^ = 0.33 Å) and 4.25 Å (Δ*d*^+^ = 0.75 Å) in Glu–His–Arg and Glu–His–Lys, respectively, and *ᾱ*^–^ is reduced to 66.0° (Δ*ᾱ*^+^ = –11.5°) and 64.0° (Δ*ᾱ*^+^ = –13.5°), respectively. We studied in detail this phenomenon by partitioning the Asp/Glu–His contacts in such interactions into contiguous and noncontiguous contacts with respect to the amino acid sequence. Surprisingly, no synergistic effect appears in contiguous contacts, compared upon formation of triads (Δ*d*^+^ = 0.05 Å and Δ*ᾱ*^+^ = –2.3°), thus reinforcing the hypothesis of a strong cooperative energy beyond structural and geometric hindrance. Second, the Asp–Phe anion–π interaction favors ternary complexes with Arg rather than Lys, suggesting that the cooperativity effects in the former triad are of a greater extent. This hypothesis is also supported by an increase in *ᾱ*^–^ (Δ*ᾱ*^–^ = 7.9°). Third, we detect a significant accumulation of Glu–Trp–(Arg/Lys) compared to the parent Asp triads, suggesting that in such complexes Trp has a preference to interact with Glu instead of Asp (Table 3).

#### 2.4.2. Anion–π–cation in protein surfaces

Protein–protein interactions (PPIs) are involved in a wide range of biological processes within the cell, including signal transduction and allosteric regulation of enzymes, through intricate networks of strong and weak transient interactions.^53^ Hence, understanding the physical relations between proteins is of pivotal importance to comprehend the molecular mechanisms of cell regulation at the atomic level. Remarkably, we identified hundreds of anion–π–cation contacts in inter-chain surfaces (Table 4 and ESI Table 20†). Compared with the ones obtained from the general search, Glu is more present than Asp (ESI Table 19†), and the abundance of Phe has been increased by *ca.* 25% up to 43.2% in detriment of His and Tyr, while the relative amounts of Arg and Lys are kept more or less constant. Glu–Phe–Arg and Asp–Phe–Arg are the most numerous triads, accounting for 34.1% of all contacts (only 15.6% in the general ternary protein search, Table 3) and representing 78.9% and 47.9% of Phe and Arg, respectively. Therefore, the anion–Phe–Arg recognition motif seems to play a very important role in inter-chain interactions. It is worth mentioning, too, the abundance of Glu–Trp–Lys. Another point is that Glu and Asp have different preferences for anion–π interaction with aromatic amino acids when only inter-chain interfaces are considered: the percentages of the interaction of Glu with Phe and Trp are bigger, in detriment of the interactions with Tyr. The amount of Asp in the anion–π interactions with Tyr, Trp, and especially His decreases in benefit of the interaction with Phe, which is dramatically increased to become the most important amino acid. The geometrical parameters of triads in interfacial interactions are similar to those obtained from the general ternary search.

**Table 4:** The most common ternary anion–π–cation surface interactions in proteins. Triads of interacting residues and their occurrences in number (amount), percentage (%), and residues' representativities for each distinct anion (%A^–^), central π-system (%π_c_), and cation (%C^+^). The expected amount of each interaction pair, according to its relative abundance, and the statistical significance are shown (Methods). Statistical significance is denoted with * for *p*-value < 0.05

Interaction	Amount (*expected*)	%	%A^–^	%π_c_	%C^+^
Glu–Phe–Arg	47 (46)	17.8	31.3	41.2	25.0
Asp–Phe–Arg	43 (35)	16.3	37.7	37.7	22.9
Asp–Tyr–Arg	21 (18)	8.0	18.4	35.6	11.2
Glu–His–Arg	21 (17)	8.0	14.0	48.8	11.2
Glu–Trp–Lys	20 (8)*	7.6	13.3	41.7	26.3
Glu–Trp–Arg	20 (19)	7.6	13.3	41.7	10.6
Glu–Tyr–Arg	19 (24)	7.2	12.7	32.2	10.1
Asp–Phe–Lys	14 (14)	5.3	12.3	12.3	18.4
Asp–His–Arg	10 (13)	3.8	8.8	23.3	5.3
Glu–Phe–Lys	10 (19)	3.8	6.7	8.8	13.2

**Fig. 6:**
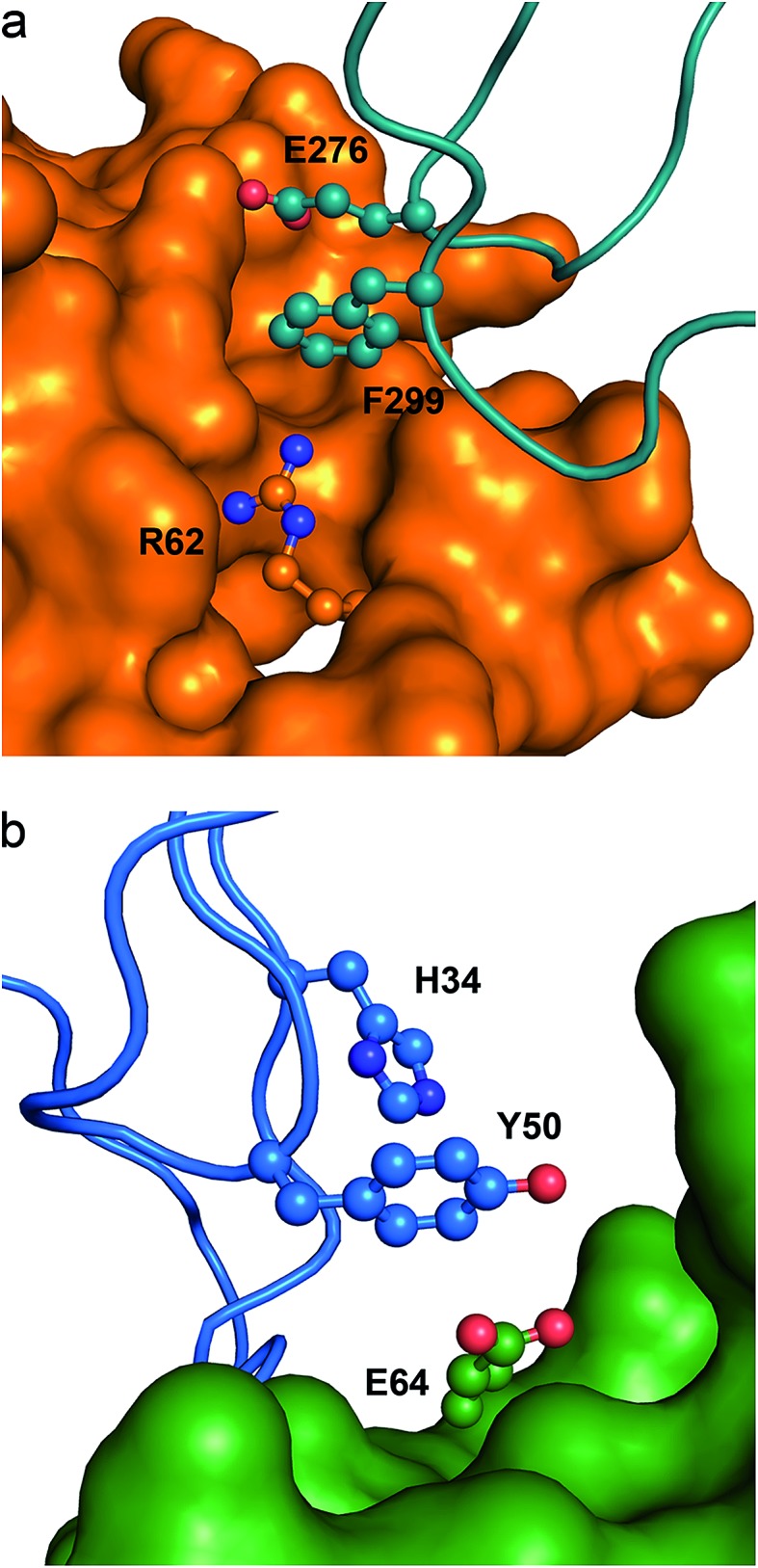
Ternary anion–π interactions in protein–protein complexes. (a) Human PRMT5 (orange surface) interacting with MET50 (turquoise ribbon) (PDB code 4GQB), where a Glu–π–Arg contact at the protein–protein interface is shown. (b) Human IL-1β (green surface) interacting with the antibody canakinumab (blue ribbon) (PDB code ; 4G6J), where a Glu–π–π contact at the protein–protein interface is shown.

In Fig. 6a we show an example of an anion–π–cation interaction occurring at the interface of the protein arginine methyltransferase 5 (PRMT5) in contact with methylosome protein 50 (MEP50).^54^ PRMT5 symmetrically di-methylates the two-terminal ω-guanidino nitrogens of arginine residues on substrate proteins, including histone tails, hence it is involved in cell signaling and gene regulation. The function and specificity of PRMT5 is regulated by a multimeric complex, a core component of which is MEP50. The figure illustrates that Glu276 from MEP50 is engaged in an intra-molecular anion–π interaction with Phe299, which in turn is recognized by Arg62 at the surface of PRMT5. Therefore the resulting anion–π–cation triad at the interface of these two proteins may play a role in their mutual recognition and the subsequent signal transduction process.

Other interesting examples for the anion–π–cation triad in protein surfaces include the contact between E443B–F252A–R413A in α-glucosidase (AglA, PDB code 1OBB), E246B–W298A–R245D in adenylosuccinate lyase (; 1DOF), D610B–Y611B–R297A in d-alanine:d-lactate ligase (; 1EHI), E91F–Y21G–K179F in green fluorescent protein (; 2C9I), and D317B–Y231A–R215A in the human glucuronyltransferase GlcAT-S (; 2D0J).

#### 2.4.3. Anion–π–π

The search for anion–π–π interactions in proteins returned 2945 successful hits out of 82 456 anion–π interactions, meaning that 3.6% of the anion–π interacting aromatic systems are involved in π–π interactions (Table 5 and ESI Tables 21 and 22†). Although the relative amount of anions and the weights of the central aromatic moieties are similar to those found for the parent binary interactions (ESI Table 6†), it is worth noting that the decrease and increase of central His and Trp is *ca.* 7%, respectively, Tyr (32.1%) being the most frequent central amino acid. If we consider separately the anion–π contacts by amino acid we observe the following results: when His is the central amino acid, the most abundant anion is Asp (53.4%). However, Glu is the most abundant anion when interacting with Phe (58.8%), Trp (57.1%), and especially Tyr (63.2%). The side chain of Phe is the most common terminal aromatic system involved in π–π interactions, accounting for almost 50% of these interactions, followed by Tyr (23.5%), His (16.9%), and Trp (15.0%).

**Table 5:** The most common ternary anion–π–π interactions in proteins. Triads of interacting residues and their occurrences in number (amount), percentage (%), and residues' representativities for each distinct anion (%A^–^), central (%π_c_), and terminal (%π_t_) π-systems. The expected amount of each interaction pair, according to its relative abundance, and the statistical significance are shown (Methods). Statistical significance is denoted with *** for *p*-value < 0.001

Interaction	Amount (*expected*)	%	%A^–^	%π_c_	%π_t_
Glu–Tyr–Phe	409 (237)***	13.9	24.7	43.3	31.1
Glu–Phe–Tyr	194 (103)***	6.6	11.7	25.0	28.0
Glu–Phe–Phe	170 (195)	5.8	10.3	21.9	12.9
Asp–Phe–Phe	148 (143)	5.0	12.2	19.1	11.3
Asp–Tyr–Phe	146 (174)	5.0	12.0	15.5	11.1
Asp–His–His	117 (49)***	4.0	9.6	16.7	23.5
Glu–Tyr–Tyr	112 (125)	3.8	6.8	11.9	16.2
Asp–His–Phe	111 (128)	3.8	9.1	15.8	8.4
Asp–Trp–Phe	110 (97)	3.7	9.0	21.0	8.4
Glu–His–Phe	104 (175)***	3.5	6.3	14.8	7.9

**Fig. 7:**
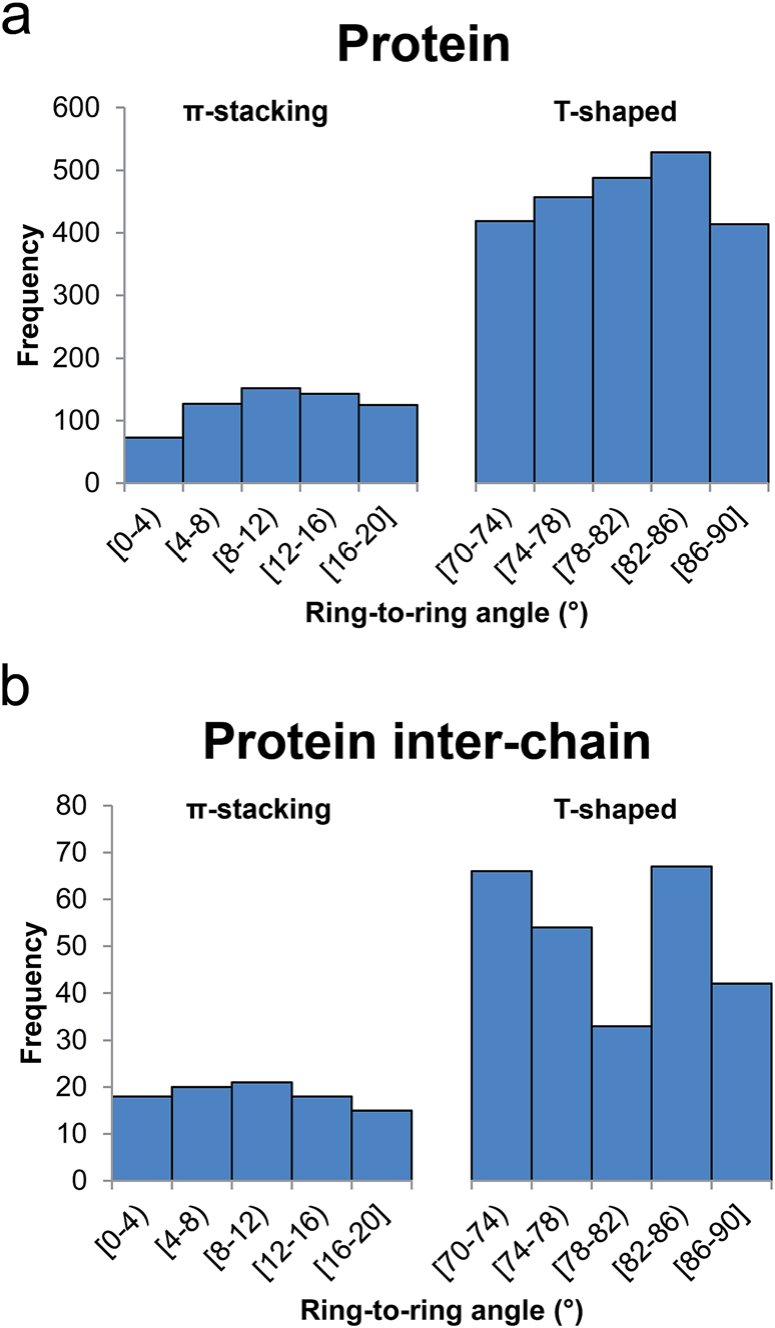
Histograms of ring-to-ring angle for ternary anion–π–π interactions in (a) proteins and (b) protein inter-chain interfaces.

In Fig. 7a we show a histogram of the ring-to-ring angle of the π–π interactions in proteins, a geometrical parameter that gives information regarding the relative orientation of the terminal aromatic ring with respect to the central π-system. The histogram reveals two well-defined, asymmetrically represented states which can be easily associated with π-stacking (from 0° to 20°) and T-shaped (from 70° to 90°) interactions (Fig. 1). Remarkably, and in contrast to anion–π–π triads involving nucleic acids, the T-shaped interaction accounts for 78.8% of contacts. In addition, if we dissect the incidence of the T-shaped and π-stacking interaction depending upon the aromatic side chain of the amino acids, interesting results are found: the ring less involved in T-shaped interactions is the imidazole moiety of His (54.2%), despite its higher polarity (Fig. 4), in favor of the phenyl moiety of Phe (87.5%). The results for Trp and Tyr are 80.0% and 79.4%, very close to the mean value of 78.8%.

From the inspection of the results in Table 5 it is worth emphasizing that Glu–Tyr–Phe is by far the most frequent recognition pattern, representing 14% of all triads. It involves almost half (43.3%) of Tyr in anion–π interactions, 31.1% of the terminal π–π interacting Phe, and 24.7% of Glu. We calculated the expected abundance and significance of each triad (Methods). As inferred from the results, there is a significant enrichment of Glu–Tyr–Phe, Glu–Phe–Tyr, Glu–Trp–His, Glu–Trp–Trp, Asp–His–Trp, and Asp–His–His, that is to say, on the one hand those triads where the pairs between Tyr and Phe are interacting with Glu and, on the other hand, those where all combinations of His and Trp are interacting with Asp and Glu as above mentioned: Glu with Trp and Asp with His. On the contrary, we observe a significant underrepresentation of other combinations, namely Glu–His–Phe, Glu–Trp–Phe, Glu–Tyr–His, Glu–Tyr–Trp, Glu–Phe–Trp, and Asp–Tyr–His.

Comparing the results of the binary interaction (Table 2) with those of the ternary contacts shows that Glu and Asp do not have the same preference for the aromatic rings to establish anion–π interactions. Moreover, the relative weight of each anion–π contact has changed: the percentages of the interaction of Glu with Trp and Tyr are bigger in the ternary search, in detriment of the percentage of the interaction with His, which dramatically decreases from 29.8% to 18.4%. For Asp, the amount of Asp–Trp also increases but, in this case, in detriment of the Asp–Phe contact. Phe and Tyr contribute largely to π–π contact pairs: a very remarkable 60.5% and 43.5% of π_c_ Tyr and π_t_ Phe, respectively, are found in the most numerous Tyr–Phe pair. Additionally, Phe–Phe and Phe–Tyr gather 41.7% and 39.6% of the central Phe and terminal Tyr, respectively. Substantial amounts of central Phe (35.4%), Trp (36.1%), and His (32.7%) and terminal His (36.4%) and Trp (32.2%) are also found in the Phe–Tyr, Trp–Phe, His–Phe, His–His, and His–Trp, respectively.

The analysis of the geometrical interaction parameters yields a *d*^–^ value (4.33 Å) slightly shorter than that reported for the binary search in proteins (4.36 Å), suggesting that the π–π interaction slightly favors the anion–π interaction. If we break down the results in terms of amino acid contributions, interesting results arise. First, His is the only amino acid that does not suffer variations in *d*^–^ (4.16 Å for both binary and ternary searches). For the rest of the amino acids, *d*^–^ values in the ternary search are shorter than those in the binary search (from 4.41 Å to 4.37 Å for Phe, from 4.40 Å to 4.30 Å for Trp, and from 4.44 Å to 4.37 Å for Tyr). The fact that the π–π interaction slightly favors the anion–π interaction for all amino acids but His could be due to dispersion effects, the contribution of which is larger in π–π interactions involving bigger, more polarizable arenes.^55^ Another aspect worth noting is that there are differences in *d*^–^ depending on whether π_t_ is engaged in π-stacking (4.20 Å) or T-shaped (4.35 Å) interactions, regardless of the anion and the central aromatic ring.

The *d*^π–π^ and ring-to-ring angles have to be separated into two different classes: one for the π-stacking (3.80 Å and 10.9°) and the other one for the T-shaped (5.09 Å and 80.3°) interactions. Very subtle differences are observed if we break down the results in terms of amino acids. For instance, the smallest/largest values of the median π-stacking (T-shaped) distances and ring-to-ring angles are 3.72 Å/3.93 Å (5.00 Å/5.13 Å) and 8.0°/11.2° (78.6°/81.3°) for His/Phe (Trp/Phe) and Trp/His (Phe/Tyr), respectively.

#### 2.4.4. Anion–π–π in protein interfaces

We also collected the inter-chain contacts in proteins and compared them with those retrieved from the general ternary search (Table 6 and ESI Tables 23 and 24†). The percentage of Glu and Asp, and of the central aromatic amino acids is very similar (ESI Table 21†). However, the abundance of the terminal amino acids varies: the content in Phe increases to 53.7% (ΔPhe = 9.1%), in detriment of the content in Tyr (ΔTyr = –3.5%) and Trp (ΔTrp = –5.4%).

**Table 6:** The most common ternary inter-chain anion–π–π interactions in proteins. Triads of interacting residues and their occurrences in number (amount), percentage (%), and residues' representativities for each distinct anion (%A^–^), central (%π_c_), and terminal (%π_t_) π-systems. The expected amount of each interaction pair, according to its relative abundance, and the statistical significance are shown (Methods). Statistical significance is denoted with ** for *p*-value < 0.01, and *** for *p*-value < 0.001

Interaction	Amount (*expected*)	%	%A^–^	%π_c_	%π_t_
Glu–Tyr–Phe	46 (34)	13.0	22.4	42.2	24.2
Asp–Trp–Phe	41 (15)***	11.6	27.5	63.1	21.6
Glu–Phe–Phe	38 (30)	10.7	18.5	39.2	20.0
Glu–His–His	23 (8)**	6.5	11.2	27.7	39.0
Asp–Phe–Phe	22 (22)	6.2	14.8	22.7	11.6
Asp–Tyr–Phe	21 (25)	5.9	14.1	19.3	11.1
Glu–Phe–Tyr	20 (11)	5.6	9.8	20.6	28.2
Glu–Tyr–Tyr	14 (13)	4.0	6.8	12.8	19.7
Glu–His–Tyr	12 (10)	3.4	5.9	14.5	16.9
Asp–His–Trp	10 (3)	2.8	6.7	12.0	29.4

In general, we observe that Glu and Asp have different preference to interact *via* anion–π with the aromatic amino acids: the percentages of the interaction of Glu with His and Phe are bigger in protein interfaces, in detriment of the percentage of the interaction with Trp. Conversely, for Asp the anion–π interactions with His, Phe, and Tyr decrease in benefit of the interaction with Trp, which becomes the most important amino acid. Regarding the π–π contact pairs, Tyr–Phe is the most numerous pair, just as in the general ternary search. The main differences appear in the π–π contact pairs formed by either Trp or His: Phe–Trp was not detected and Trp–His (3 hits) is rare. On the other hand, Trp–Phe and His–His are quite abundant with important contributions of central Trp (72.3%) and terminal His (54.2%).

The comparison of the geometrical parameters of anion–π–π contacts in proteins and in peptide surfaces revealed a longer *d*^–^ value for the latter (*d*^–^ = 4.45 Å, Δ*d*^–^ = 0.12 Å). His exhibits the shortest *d*^–^ (4.19 Å), as in the general search, followed by Phe (4.40 Å), Trp (4.50 Å), and Tyr (4.70 Å). In contrast to the tendency observed in the previous search, the differences in *d*^–^ depending on whether the terminal aromatic ring is engaged in π-stacking or T-shaped interactions are very small. However, the histogram represented in Fig. 7b shows that, also in contrast to the general search, the interface T-shaped interactions are unevenly distributed along the considered angles: the central bin, comprising 78–82°, contains 33 hits, whereas the 70–74° and 82–86° bins each contain double that amount (66 and 67 hits, respectively).

The statistical analysis of the respective abundances sheds light on the characteristic preferences among amino acids when interacting at protein interfaces (Table 6 and ESI Table 24†). First, there is a remarkable enrichment in Asp–Trp–Phe (41 hits out of 110 contacts in proteins, Table 5), *i.e.* 37.3% of these interactions occur between amino acids of different peptide chains. As we identified 191 Asp–Trp binary interactions in peptide surfaces (ESI Table 9†), 21.5% of them are involved in this triad. This enrichment is compensated by a significant loss of the tandem triad Glu–Trp–Phe (6 hits), which is also underrepresented in the general search. Second, there is an enrichment of Glu–His–His as an inter-chain contact as well (23 hits, 37.1% of all contacts in proteins). Intriguingly, the corresponding Asp ternary complex is underrepresented at the interfaces of proteins (9 hits) despite being over represented in the general search (117 hits, Table 5). Consistently, the inter-chain anion–π interaction between the His dimer and Glu is remarkably shorter than that with Asp (Δ*d*^–^_π-stacking_ = –0.54 Å), suggesting a strong anion-specific cooperativity effect. Third, the binary interactions (Asp/Glu)–His weaken upon forming ternary complexes with Phe: on the one hand they are significantly underrepresented (ESI Table 24†); on the other hand there is an increase in *d*^–^ upon formation of the triads, particularly for Glu (Δ*d*^–^_Asp_ = 0.06 Å, Δ*d*^–^_Glu_ = 0.32 Å). Hence, not all combinations of anions and aromatic side chains that form triads in proteins present cooperativity effects. Consistently, those triads appear rarely in crystal structures. Last, the results for the Glu–Trp–His triad are especially striking: it is enriched in the general ternary protein search (92 hits, Table 5), and yet it is underrepresented at the interfaces (1 hit), *i.e.* less than 0.5% of surface Glu–Trp pairs are involved in ternary interactions with His (231 hits, ESI Table 9†).

Fig. 5c depicts a snapshot of the active site of the *Plasmodium falciparum* glutathione *S*-transferase (PfGST).^56^ GSTs catalyze the conjugation of glutathione with a wide variety of hydrophobic compounds, generally resulting in nontoxic products that can be readily eliminated. PfGST is highly abundant in the parasite, its activity has been found to be increased in chloroquine-resistant cells, and it has been shown to act as a ligandin in parasitotoxic hemin. Thus, the enzyme represents a promising target for antimalarial drug development. In the figure we observe an anion–π interaction between Glu120 from one monomer and the phenyl side chain of Phe10 from a second monomer. Furthermore, Phe35 interacts *via* a T-shaped intra-chain contact with Phe10 giving rise to an anion–π–π interaction. Formate 1, that presumably mimics the glycyl carboxylate of glutathione, is interacting with Glu120 suggesting that this Glu, that is entangled in an anion–π interaction, might be important for the catalytic activity of the enzyme. Additionally, formate 2 presumably mimics the glutamyl carboxylate of glutathione. In this example, the anion–π–π interaction might not only provide functional assistance, but it could also be important for the successful crystallization process of the protein as it stabilizes its dimeric form.

In Fig. 6b we show a second example of an anion–π–π interaction involved in protein–protein recognition, which occurs at the interface of interleukin-1β (IL-1β) in contact with the highly specific IL-1β monoclonal antibody canakinumab.^57^ IL-1β is a key orchestrator in inflammatory and immune responses forming a heterotrimeric signaling-competent complex with IL-1-specific receptor proteins. The antibody neutralizes the signal transduction by reducing the affinity of IL-1β for the complex in a competitive inhibitory manner. The figure illustrates that Glu64 from IL-1β is engaged in an anion–π interaction with Tyr50 from the antibody. The binary interaction is cooperatively strengthened by forming a triad with His34 in a T-shaped π–π contact. Thus, the interfacial anion–π–π interaction contributes to the recognition process and the stabilization of the IL-1β:canakinumab complex.

## 3 Conclusions

Thousands of anion–π contacts have been detected from a large-scale analysis of the protein data bank, revealing selectivities among anions, cations, and π-systems not yet reported in a biological context. Due to their abundance, Asp and Glu are found in the vast majority of anion–π interactions with a preferred close-to-parallel orientation of their carboxylate with respect to the interacting aromatic system. For nucleic acids different results are obtained: in DNA there is no selectivity towards either Glu or Asp whereas Glu is more present in RNA. In addition Asp is prone to interact with cytosine and thymine and Glu with thymine in DNA, whereas in RNA Asp and Glu prefer cytosine and adenine, respectively. Anion–π distances also show different trends, since they are shorter for Glu than for Asp in DNA whereas the opposite is observed in RNA. For proteins, a very remarkable 61.3% of all processed PDB structures present anion–π interactions, where Glu is the major anion and His the most common amino acid. However, at inter-chain contacts and protein–protein interfaces Tyr is more abundant than His at the expense of Trp. Importantly, the anion–π recognition pattern in proteins varies when considering only inter-chain interactions.

Remarkably, hundreds of cation–π, π-stacking, and T-shaped interactions have been observed on the opposite side of the aromatic ring involved in anion–π interactions, a fact that might lead to cooperativity effects. Concerning anion–π–cation interactions in RNA, the Glu–A–Arg triad represents 87% of all contacts, whereas in proteins, Glu and Asp have very similar contributions and Arg is the most abundant cation (69%). When only inter-chain contacts are considered, the anion–Phe–Arg pattern predominates. In anion–π–π interactions Asp is more abundant in nucleic acids in contraposition to the binary search, with Asp–His–T and Asp–C–A as the major contributors in DNA and RNA, respectively. In proteins, if π–π interactions are taken into account different anion–π recognition patterns are obtained when considering binary or ternary contacts: Tyr and Phe are the most abundant π-systems involved in anion–π and π–π interactions, respectively, which leads to Glu–Tyr–Phe being the most abundant triad. In peptide interfaces we have detected a significant enrichment of Asp–Trp–Phe. We have also observed that T-shaped interactions are much more abundant than π-stacking interactions in proteins and that the anion–π equilibrium distance in triads is slightly shorter than that of the binary contacts, suggesting that the π–π interaction favors the anion–π interaction.

The reported results bring striking conclusions: overall, more than half of the biomolecular complexes analyzed contained at least one anion–π contact. In other words, there is one anion–π interaction for every 50 anionic residues in the PDB. Additionally, thousands of them were engaged in triads. Hence, anion–π interactions and the cooperativity that arises from ternary contacts are a common resource in molecular science, and may play an active role in protein folding and function, and nucleic acids–protein and protein–protein recognition by making a significant contribution to the binding energy of protein complex formation and stabilization. Besides the mentioned biological roles, we present here examples of anion–π interactions and related triads involved in enzymatic catalysis, epigenetic gene regulation, antigen–antibody recognition, and protein crystallography.

## 4 Methods

### 4.1. Data collection

A multi-processor Python routine using Biopython^58^ was designed to process the whole PDB database, identify the interactions of interest, and keep a record of any hits in a two-step manner: initially, each available PDB structure solved by X-ray crystallography or neutron diffraction with a resolution higher than 2.5 Å (62 033 out of 89 395 PDB structures) was queried for anion–π interactions taking into consideration the distance and the angle between the partners (ESI Table 1† for a comprehensive definition of centers and centroids for each amino acid, nucleic base, and ion taken into account). Resulting binary interactions were subsequently queried for cations or aromatic systems in close proximity in order to gather tertiary complexes, *i.e.* anion–π–cation and anion–π–π. Aromatic stacking interactions were considered in face-to-face, T-shaped, and parallel-displaced configurations. To reduce the number of redundant interactions found in different chains of the same structure, binary and ternary interactions arising from the same residue names and numbers in a PDB file were omitted.

### 4.2. Statistical analysis

The expected amount of each interaction was computed using the observed abundance of each partner in the interaction rounded to the closest integer, *e.g.* the expected amount of Glu–DT binary anion–π interactions in DNA (ESI Table 3†) was computed as:
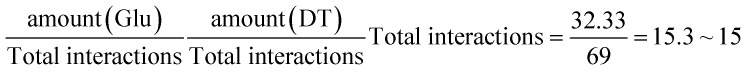


The statistical significance of the difference between the expected and the observed amounts for each interaction was assessed by means of the Fischer's exact test of the corresponding contingency tables, as implemented in the statistical package R v3.0.^59^ Statistical significance is denoted in the manuscript with * for *p*-value < 0.05, ** for *p*-value < 0.01, and *** for *p*-value < 0.001.

## Supplementary Material

Supplementary information

Supplementary information
