# Supplementary material for: A thorough anion–π interaction study in biomolecules: on the importance of cooperativity effects
Source: Chem Sci. 2015 Jun 5;7(2):1038–50. doi: 10.1039/c5sc01386k (PMC5967298; doi:10.1039/c5sc01386k)
Supplement: Supplementary file 2 [file SC-007-C5SC01386K-s002.pdf]

Electronic Supplementary Material (ESI) for Chemical Science.
This journal is © The Royal Society of Chemistry 2015

SUPPORTING INFORMATION
FOR

"A Thorough Anion–π Interaction Study in Biomolecules: On the Importance of Cooperativity Effects"

Xavier Lucas,[1] Antonio Bauzá,[2] Antonio Frontera,[2] and David Quiñonero,*[2]*

[1] Pharmaceutical Bioinformatics, Institute of Pharmaceutical Sciences, Albert-Ludwigs-University, Hermann-Herder-Str. 9, D-79104 Freiburg, Germany.

[2] Departament de Química, Universitat de les Illes Balears, Crta. de Valldemossa km 7.5, 07122 Palma de Mallorca, Spain.

* e-mail: david.quinonero@uib.es, Phone: +34 971173498, Fax: +34 971173426; e-mail: xavier.lucas@pharmazie.uni-freiburg.de

INDEX

-Supplementary Figures 1 and 2

-Supplementary Tables 1–24

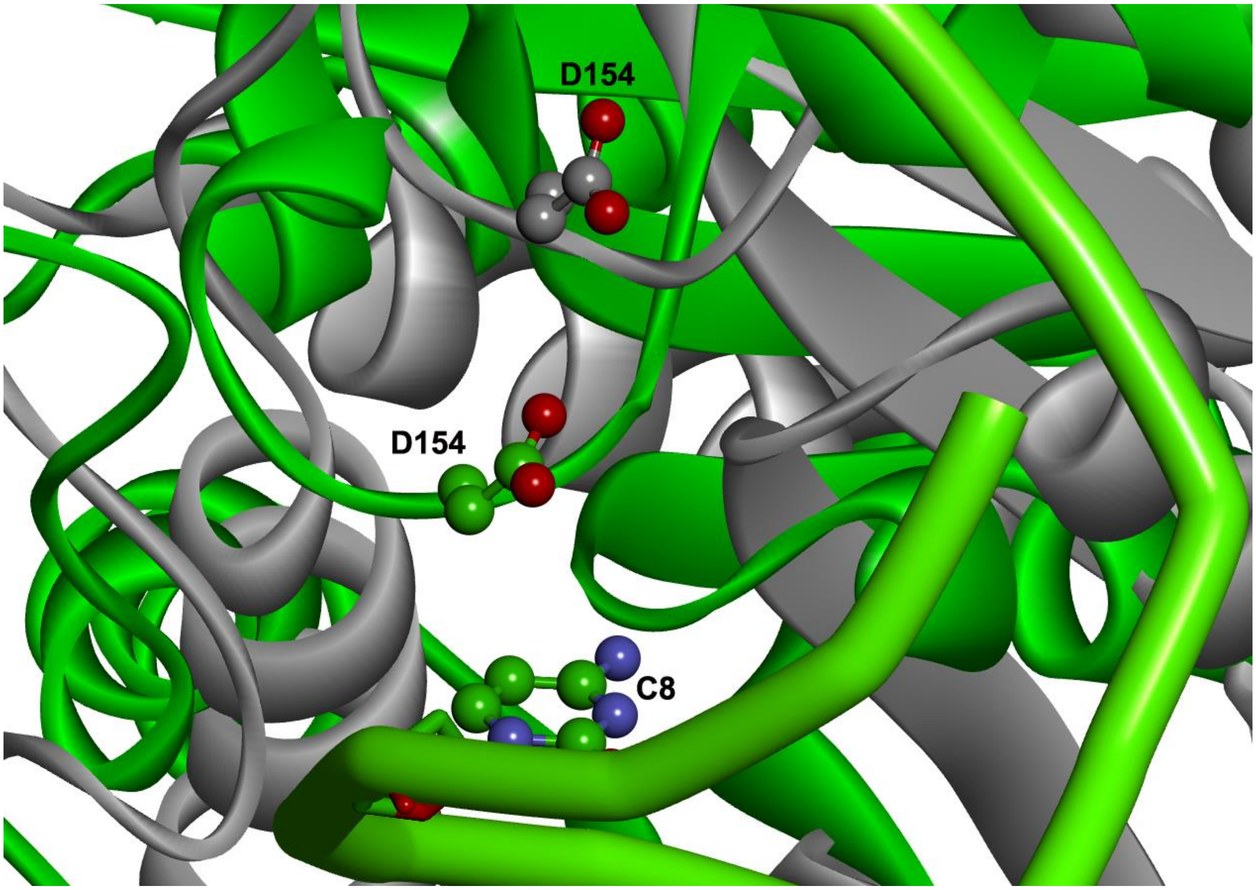

Supplementary Figure 1. Overlay of the *Bam*HI type II restriction endonuclease in its *apo* form (PDB code 1BAM, in grey) and in the pre-reactive state bound to DNA (PDB code 2BAM, in green), where the displacement of Asp154 is patent upon DNA binding.

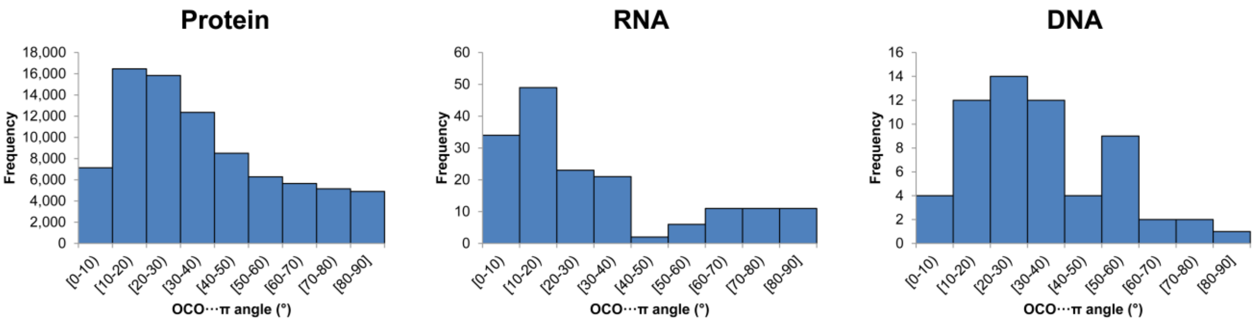

Supplementary Figure 2. Histograms of carboxylate–π angle protein, RNA, and DNA binary anion–π interactions for Asp and Glu.

Supplementary Table 1. Definition of centers and centroids for the different amino acids, nucleic bases, and ions considered during data collection. For polyatomic molecules, considered atom coordinates used to extract the corresponding centroid are highlighted.

Aromatic systems				
DNA	DA 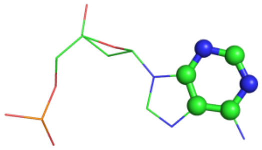	DG 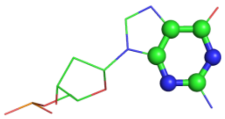	DC 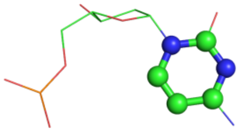	DT 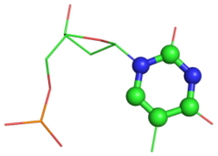
RNA	A 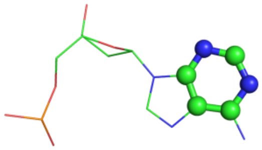	G 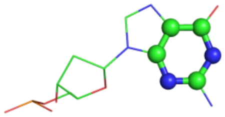	C 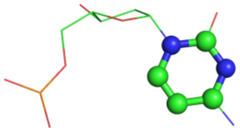	U 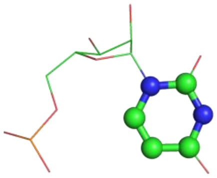
Protein	HIS 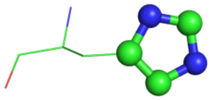	PHE 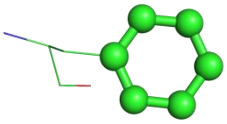	TRP 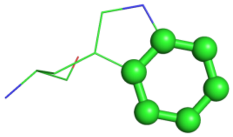	TYR 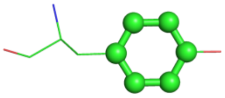

Anions			
ASP 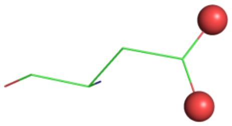	GLU 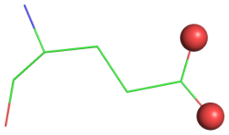	SO4 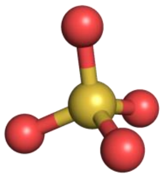	F<br>CL<br>BR<br>IOD
CO3 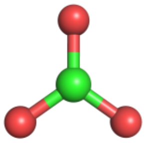	NO3 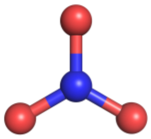	PO4 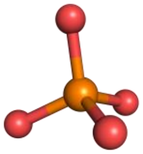	

Cations		
ARG 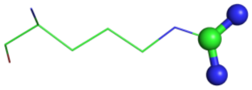	LYS 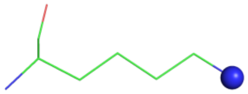	NA<br>K<br>CA<br>MG

Supplementary Table 2. Interacting residues from the search of anion–π interactions in DNA, their amounts, and their respective percentages within the anion and deoxynucleotide (in italics) groups.

Interacting residues	Amount	%
DT	33	*47.8*
Glu	32	46.4
Asp	31	44.9
DC	29	*42.0*
DA	6	*8.7*
SO_4	5	7.2
Cl	1	1.4
DG	1	*1.4*

Supplementary Table 3. Binary anion–π interactions in DNA. Pairs of interacting residues and their occurrences in number (amount), percentage (%), and residues' representativities for each distinct anion (%A^-) and π-system (%π). The expected amount of each interaction pair, according to its relative abundance, is shown. No statistically significant enrichments are found (Methods).

Interaction	Amount (*expected*)	%	%A^-	%π
Glu–DT	18 (*15*)	26.1	56.2	54.5
Asp–DC	16 (*13*)	23.2	51.6	55.2
Asp–DT	14 (*15*)	20.3	45.2	42.4
Glu–DC	9 (*13*)	13.0	28.1	31.0
Glu–DA	5 (*3*)	7.2	15.6	83.3
SO_4–DC	4 (*2*)	5.8	80.0	13.8
SO_4–DT	1 (*2*)	1.4	20.0	3.0
Asp–DA	1 (*3*)	1.4	3.2	16.7

Cl–DG	1 (*0*)	1.4	100.0	100.0

Supplementary Table 4. Binary anion–π interactions in RNA. Pairs of interacting residues and their occurrences in number (amount), percentage (%), and residues' representativities for each distinct anion (%A^-) and π-system (%π). The expected amount of each interaction pair, according to its relative abundance, and the statistical significance are shown (Methods).

**Interaction**	**Amount (*expected*)**	**%**	**%A^-**	**%π**
Glu–A	64 (*43*) *	32.5	52.0	92.8
Asp–C	40 (*12*) ***	20.3	72.7	90.9
Glu–G	30 (*29*)	15.2	24.4	65.2
Glu–U	27 (*24*)	13.7	22.0	71.1
Cl–G	13 (*3*) *	6.6	100.0	28.3
Asp–U	9 (*11*)	4.6	16.4	23.7
Asp–A	4 (*19*) **	2.0	7.3	5.8
Asp–G	2 (*13*) **	1.0	3.6	4.3
SO_4–C	2 (*1*)	1.0	33.3	4.5
Glu–C	2 (*27*) ***	1.0	1.6	4.5
SO_4–U	2 (*1*)	1.0	33.3	5.3
SO_4–G	1 (*1*)	0.5	16.7	2.2
SO_4–A	1 (*2*)	0.5	16.7	1.4

Supplementary Table 5. Interacting residues from the search of anion–π interactions in RNA, their amounts, and their respective percentages within the anion and nucleotide (in italics) groups.

Interacting residues	Amount	%
Glu	123	62.4
A	69	*35.0*
Asp	55	27.9
G	46	*23.4*
C	44	*22.3*
U	38	*19.3*
Cl	13	6.6
SO_4	6	3.0

Supplementary Table 6. Interacting residues from the search of anion–π interactions in proteins, their amounts, and their respective percentages within the anion and amino acid (in italics) groups.

Interacting residues	Amount	%
Glu	46,132	55.9
Asp	34,214	41.5
His	24,677	*29.9*
Phe	24,035	*29.1*
Tyr	24,003	*29.1*
Trp	9,741	*11.8*
SO_4	1,055	1.3
Cl	627	0.8
PO4	261	0.3
NO3	94	0.1
CO3	46	0.1

Br	26	0.0
F	1	0.0

Supplementary Table 7. Binary anion–π interactions in proteins. Pairs of interacting residues and their occurrences in number (amount), percentage (%), and residues' representativities for each distinct anion (%A$^-$) and π-system (%π). The expected amount of each interaction pair, according to its relative abundance, and the statistical significance are shown (Methods).

Interaction	Amount (*expected*)	%	%A$^-$	%π
Glu–His	13,763 (*13,805*)	16.7	29.8	55.8
Glu–Tyr	13,592 (*13,428*)	16.5	29.5	56.6
Glu–Phe	13,060 (*13,446*) **	15.8	28.3	54.3
Asp–Phe	10,477 (*9,972*) ***	12.7	30.6	43.6
Asp–His	10,180 (*10,239*)	12.3	29.8	41.3
Asp–Tyr	9,797 (*9,959*)	11.9	28.6	40.6
Glu–Trp	5,717 (*5,450*) **	6.9	12.4	58.7
Asp–Trp	3,760 (*4,042*) **	4.6	11.0	38.6
SO_4–His	405 (*316*) **	0.5	38.4	1.6
SO_4–Tyr	274 (*307*)	0.3	26.0	1.1
SO_4–Phe	253 (*308*) *	0.3	24.0	1.1
Cl–Tyr	193 (*182*)	0.2	30.8	0.8
Cl–His	183 (*188*)	0.2	29.2	0.7
Cl–Phe	163 (*183*)	0.2	26.0	0.7
SO_4–Trp	123 (*125*)	0.1	11.7	1.3
PO_4–His	110 *(78)* *	0.1	42.1	0.4
Cl–Trp	88 (*74*)	0.1	14.0	0.9
PO_4–Tyr	70 *(76)*	0.1	26.8	0.3
NO_3–Tyr	51 *(27)* **	0.1	54.3	0.2
PO_4–Phe	47 *(76)* *	0.1	18.0	0.2
PO_4–Trp	34 *(31)*	0.0	13.0	0.3

CO_3–Tyr	23 *(13)*	0.0	50.0	0.1
NO_3–Phe	23 *(27)*	0.0	24.5	0.1
NO_3–His	15 *(28)*	0.0	16.0	0.1
Br–His	13 (*8*)	0.0	50.0	0.1
CO_3–Trp	9 *(5)*	0.0	19.6	0.1
CO_3–His	7 *(14)*	0.0	15.2	0.0
CO_3–Phe	7 *(13)*	0.0	15.2	0.0
Br–Phe	5 (*8*)	0.0	19.2	0.0
Br–Trp	5 (*3*)	0.0	19.2	0.1
NO_3–Trp	5 *(11)*	0.0	5.3	0.1
Br–Tyr	3 (*8*)	0.0	11.5	0.0
F–His	1 (*0*)	0.0	100.0	0.0

Supplementary Table 8. All interacting residues studied in our working set of 62,033 PDB structures, their amounts, and their respective percentages within the anion, cation (in bold), amino acid (in italics), nucleotide (underlined), and deoxynucleotide (underlined and in italics) groups.

**Interacting residues**	**Amount**	**%**
Glu	2,121,596	51.7
Asp	1,918,809	46.8
Lys	1,816,877	**52.0**
Arg	1,631,104	**46.7**
Phe	1,349,690	*35.2*
Tyr	1,186,004	*31.0*
His	803,142	*21.0*
Trp	491,434	*12.8*
SO_4	34,818	0.8
G	21,904	31.4

DG	18,996	*28.6*
C	18,380	26.3
DC	17,350	*26.1*
Mg	17,051	**0.5**
A	16,591	23.8
Cl	16,581	0.4
Ca	16,126	**0.5**
DT	15,228	*22.9*
DA	14,859	*22.4*
U	12,975	18.6
Na	9,846	**0.3**
PO4	6,040	0.1
K	3,158	**0.1**
Br	1,397	0.0
NO3	987	0.0
CO3	342	0.0
F	48	0.0

Supplementary Table 9. Binary inter-chain anion–π interactions in proteins. Pairs of interacting residues and their occurrences in number (amount), percentage (%), and residues' representativities for each distinct anion (%A$^-$) and π-system (%π). The expected amount of each interaction pair, according to its relative abundance, is shown. No statistically significant enrichments are found (Methods).

Interaction	Amount (*expected*)	%	%A$^-$	%π
Glu–Tyr	1,162 (*1,137*)	21.5	35.7	61.6
Glu–Phe	991 (*932*)	18.4	30.5	64.1
Glu–His	868 (*928*)	16.1	26.7	56.4
Asp–Tyr	725 (*750*)	13.4	33.8	38.4

Asp–His	672 (*612*)	12.5	31.4	43.6
Asp–Phe	555 (*614*)	10.3	25.9	35.9
Glu–Trp	231 (*254*)	4.3	7.1	54.7
Asp–Trp	191 (*168*)	3.5	8.9	45.3

Supplementary Table 10. Interacting residues from the search of anion–π surface interactions in proteins, their amounts, and their respective percentages within the anion and amino acid (in italics) groups.

Interacting residues	Amount	%
Glu	3,252	60.3
Asp	2,143	39.7
Tyr	1,887	*35.0*
Phe	1,546	*28.7*
His	1,540	*28.5*
Trp	422	*7.8*

Supplementary Table 11. Ternary anion–π–π interactions in DNA. Triads of interacting residues and their occurrences in number (amount), percentage (%), and residues' representativities for each distinct anion (%A$^-$), central π-system (%π_c), and terminal π-system (%π_t). The expected amount of each interaction pair, according to its relative abundance, and the statistical significance are shown (Methods).

Interaction	Amount (*expected*)	%	%A$^-$	%π_c	%π_t
Asp–His–DT	18 (*5*) **	30.5	48.6	100.0	40.0
Glu–DT–DA	7 (*2*)	11.9	36.8	15.6	35.0
Asp–DC–DA	6 (*4*)	10.2	16.2	31.6	30.0
Asp–DC–DT	5 (*5*)	8.5	13.5	26.3	11.1
Glu–DC–DG	4 (*1*)	6.8	21.1	21.1	33.3

Asp–DT–DG	4 (*2*)	6.8	10.8	8.9	33.3
Glu–DT–DG	3 (*1*)	5.1	15.8	6.7	25.0
Asp–DT–DA	2 (*4*)	3.4	5.4	4.4	10.0
Glu–Trp–DT	2 (*0*)	3.4	10.5	100.0	4.4
Asp–Tyr–DT	1 (*0*)	1.7	2.7	100.0	2.2
Glu–DC–DA	1 (*2*)	1.7	5.3	5.3	5.0
SO_4–DT–DA	1 (*0*)	1.7	33.3	2.2	5.0
SO_4–DC–DA	1 (*0*)	1.7	33.3	5.3	5.0
Glu–DA–DA	1 (*0*)	1.7	5.3	5.0	5.0
SO_4–DC–DG	1 (*0*)	1.7	33.3	5.3	8.3
Asp–DT–DC	1 (*0*)	1.7	2.7	2.2	5.3
Glu–DT–Phe	1 (*0*)	1.7	5.3	2.2	100.0

Supplementary Table 12. Interacting residues from the search of anion–π–π interactions in DNA, their amounts (central+terminal), and their respective percentages for the anions, central, and terminal aromatic moieties.

Interacting residues	Amount	%A^-	%π_c	%π_t
DT	45 (19+26)	-	32.2	44.1
Asp	37	47.4	-	-
DA	20 (1+19)	-	1.7	32.2
DC	19 (18+1)	-	30.5	1.7
Glu	19	44.7	-	-
His	18 (18+0)	-	30.5	0.0
DG	12 (0+12)	-	0.0	20.3
SO_4	3	7.9	-	-
Trp	2 (2+0)	-	3.4	0.0
Phe	1 (0+1)	-	0.0	1.7

Tyr	1 (1+0)	-	1.7	0.0

Supplementary Table 13. Ternary anion–π–cation interactions in RNA. Triads of interacting residues and their occurrences in number (amount), percentage (%), and residues' representativities for each distinct anion (%A^-), central π-system (%π_c), and cation (%C^+). The expected amount of each interaction pair, according to its relative abundance, and the statistical significance are shown (Methods).

**Interaction**	**Amount (*expected*)**	**%**	**%A^-**	**%π_c**	**%C^+**
Glu–A–Arg	13 (*11*)	86.7	100.0	92.9	92.9
SO_4–G–Arg	1 (*0*)	6.7	100.0	100.0	7.1
Asp–A–Lys	1 (*0*)	6.7	100.0	7.1	100.0

Supplementary Table 14. Interacting residues from the search of anion–π–cation interactions in RNA, their amounts, and their respective percentages within the anion, nucleotide (in italics), and cation (in bold) groups.

**Interacting residues**	**Amount**	**%**
A	14	*93.3*
Arg	14	**93.3**
Glu	13	86.7
Asp	1	6.7
G	1	*6.7*
Lys	1	**6.7**
SO_4	1	6.7

Supplementary Table 15. Ternary anion–π–π interactions in RNA. Triads of interacting residues and their occurrences in number (amount), percentage (%), and residues' representativities for each distinct anion (%A^-), central π-system (%π_c), and terminal π-system (%π_t). The expected amount of each interaction pair, according to its relative abundance, is shown. No statistically significant enrichments are found (Methods).

Interaction	Amount (*expected*)	%	%A^-	%π_c	%π_t
Asp–C–A	12 (*5*)	46.2	70.6	85.7	92.3
Glu–His–U	5 (*0*)	19.2	71.4	100.0	83.3
Asp–U–Phe	3 (*0*)	11.5	17.6	75.0	100.0
Asp–G–A	1 (*1*)	3.8	5.9	50.0	7.7
Asp–C–C	1 (*1*)	3.8	5.9	7.1	50.0
Glu–G–Tyr	1 (*0*)	3.8	14.3	50.0	50.0
Glu–A–Tyr	1 (*0*)	3.8	14.3	100.0	50.0
SO_4–C–C	1 (*0*)	3.8	50.0	7.1	50.0
SO_4–U–U	1 (*0*)	3.8	50.0	25.0	16.7

Supplementary Table 16. Interacting residues from the search of anion–π–π interactions in RNA, their amounts (central+terminal), and their respective percentages for the anions, central, and terminal aromatic moieties.

Interacting residues	Amount	%A^-	%π_c	%π_t
Asp	17	65.4	-	-
C	16 (14+2)	-	53.8	7.7
A	14 (1+13)	-	3.8	50.0
U	10 (4+6)	-	15.4	23.1
Glu	7	26.9	-	-
His	5 (5+0)	-	19.2	0.0
Phe	3 (0+3)	-	0.0	11.5

G	2 (2+0)	-	7.7	0.0
SO_4	2	7.7	-	-
Tyr	2 (0+2)	-	0.0	7.7

Supplementary Table 17. Interacting residues from the search of anion–π–cation interactions in proteins, their amounts, and their respective percentages within the anion, amino acid (in italics), and cation (in bold) groups.

Interacting residues	Amount	%
Arg	1,652	**68.9**
Glu	1,183	49.3
Asp	1,147	47.8
Tyr	729	*30.4*
Lys	707	**29.5**
His	639	*26.6*
Phe	611	*25.5*
Trp	419	*17.5*
SO_4	44	1.8
Na	35	**1.5**
Cl	18	0.8
PO_4	5	0.2
K	4	**0.2**
NO_3	1	0.0

Supplementary Table 18. Ternary anion–π–cation interactions in proteins. Triads of interacting residues and their occurrences in number (amount), percentage (%), and residues' representativities for each distinct anion (%A^-), central π-system (%π_c), and cation (%C^+). The expected amount of each interaction pair, according to its relative abundance, and the statistical significance are shown (Methods).

Interaction	Amount (*expected*)	%	%A^-	%π_c	%C^+
Asp–His–Arg	272 (*211*) **	11.4	23.7	42.6	16.5
Asp–Tyr–Arg	268 (*240*)	11.2	23.4	36.8	16.2
Glu–Tyr–Arg	250 (*248*)	10.4	21.1	34.3	15.1
Glu–Phe–Arg	244 (*208*)	10.2	20.6	39.9	14.8
Glu–His–Arg	189 (*217*)	7.9	16.0	29.6	11.4
Glu–Trp–Arg	175 (*142*)	7.3	14.8	41.8	10.6
Asp–Phe–Arg	130 (*201*) ***	5.4	11.3	21.3	7.9
Asp–Phe–Lys	129 (*86*) **	5.4	11.2	21.1	18.2
Glu–Trp–Lys	115 (*61*) ***	4.8	9.7	27.4	16.3
Asp–Tyr–Lys	106 (*103*)	4.4	9.2	14.5	15.0
Asp–His–Lys	82 (*90*)	3.4	7.1	12.8	11.6
Asp–Trp–Arg	81 (*138*) ***	3.4	7.1	19.3	4.9
Glu–Tyr–Lys	76 (*106*) *	3.2	6.4	10.4	10.7
Glu–His–Lys	67 (*93*) *	2.8	5.7	10.5	9.5
Glu–Phe–Lys	63 (*89*) *	2.6	5.3	10.3	8.9
Asp–Trp–Lys	44 (*59*)	1.8	3.8	10.5	6.2
Asp–Phe–Na	35 (*4*) ***	1.5	3.1	5.7	100.0
SO_4–Tyr–Arg	15 (*9*)	0.6	34.1	2.1	0.9
SO_4–Tyr–Lys	9 (*4*)	0.4	20.5	1.2	1.3
SO_4–His–Arg	8 (*8*)	0.3	18.2	1.3	0.5
Cl–His–Lys	8 (*1*) *	0.3	44.4	1.3	1.1
SO_4–Phe–Lys	6 (*3*)	0.3	13.6	1.0	0.8
PO_4–His–Arg	4 (*1*)	0.2	80.0	0.6	0.2
Glu–His–K	4 (*1*)	0.2	0.3	0.6	100.0
Cl–Tyr–Arg	4 (*4*)	0.2	22.2	0.5	0.2
Cl–His–Arg	4 (*3*)	0.2	22.2	0.6	0.2
SO_4–Phe–Arg	3 (*8*)	0.1	6.8	0.5	0.2
Cl–Trp–Arg	1 (*2*)	0.0	5.6	0.2	0.1
Cl–Phe–Arg	1 (*3*)	0.0	5.6	0.2	0.1
SO_4–Trp–Lys	1 (*2*)	0.0	2.3	0.2	0.1

SO_4–His–Lys	1 (*3*)	0.0	2.3	0.2	0.1
NO_3–Trp–Arg	1 (0)	0.0	100.0	0.2	0.1
PO_4–Tyr–Arg	1 (*1*)	0.0	20.0	0.1	0.1
SO_4–Trp–Arg	1 (*5*)	0.0	2.3	0.2	0.1

Supplementary Table 19. Interacting residues from the search of anion–π–cation surface interactions in proteins, their amounts, and their respective percentages within the anion, amino acid (in italics), and cation (in bold) groups.

Interacting residues	Amount	%
Arg	188	**71.2**
Glu	150	56.8
Asp	114	43.2
Phe	114	*43.2*
Lys	76	**28.8**
Tyr	59	*22.3*
Trp	48	*18.2*
His	43	*16.3*

Supplementary Table 20. Ternary anion–π–cation surface interactions in proteins. Triads of interacting residues and their occurrences in number (amount), percentage (%), and residues' representativities for each distinct anion (%A^-), central π-system (%π_c), and cation (%C^+). The expected amount of each interaction pair, according to its relative abundance, and the statistical significance are shown (Methods).

Interaction	Amount (*expected*)	%	%A^-	%π_c	%C^+
Glu–Phe–Arg	47 (*46*)	17.8	31.3	41.2	25.0
Asp–Phe–Arg	43 (*35*)	16.3	37.7	37.7	22.9

Asp–Tyr–Arg	21 (*18*)	8.0	18.4	35.6	11.2
Glu–His–Arg	21 (*17*)	8.0	14.0	48.8	11.2
Glu–Trp–Lys	20 (*8*) *	7.6	13.3	41.7	26.3
Glu–Trp–Arg	20 (*19*)	7.6	13.3	41.7	10.6
Glu–Tyr–Arg	19 (*24*)	7.2	12.7	32.2	10.1
Asp–Phe–Lys	14 (*14*)	5.3	12.3	12.3	18.4
Asp–His–Arg	10 (*13*)	3.8	8.8	23.3	5.3
Glu–Phe–Lys	10 (*19*)	3.8	6.7	8.8	13.2
Asp–Tyr–Lys	10 (*7*)	3.8	8.8	16.9	13.2
Glu–Tyr–Lys	9 (*10*)	3.4	6.0	15.3	11.8
Asp–His–Lys	8 (*5*)	3.0	7.0	18.6	10.5
Asp–Trp–Arg	7 (*15*)	2.7	6.1	14.6	3.7
Glu–His–Lys	4 (*7*)	1.5	2.7	9.3	5.3
Asp–Trp–Lys	1 (*6*)	0.4	0.9	2.1	1.3

Supplementary Table 21. Interacting residues from the search of anion–π–π interactions in proteins, their amounts (central+terminal), and their respective percentages for the anions, central, and terminal aromatic moieties.

Interacting residues	Amount	$\%A^-$	$\%\pi_c$	$\%\pi_t$
Phe	2,089 (775+1,314)	-	26.3	44.6
Glu	1,658	56.3	-	-
Tyr	1,637 (944+693)	-	32.1	23.5
Asp	1,216	41.3	-	-
His	1,198 (701+497)	-	23.8	16.9
Trp	966 (525+441)	-	17.8	15.0
SO_4	30	1.0	-	-
Cl	23	0.8	-	-

PO_4	10	0.3	-	-
NO_3	7	0.2	-	-
CO_3	1	0.0	-	-

Supplementary Table 22. Ternary anion–π–π interactions in proteins. Triads of interacting residues and their occurrences in number (amount), percentage (%), and residues' representativities for each distinct anion (%A^-), central (%π_c), and terminal (%π_t) π-systems. The expected amount of each interaction pair, according to its relative abundance, and the statistical significance are shown (Methods).

Interaction	Amount (*expected*)	%	%A^-	%π_c	%π_t
Glu–Tyr–Phe	409 (*237*) ***	13.9	24.7	43.3	31.1
Glu–Phe–Tyr	194 (*103*) ***	6.6	11.7	25.0	28.0
Glu–Phe–Phe	170 (*195*)	5.8	10.3	21.9	12.9
Asp–Phe–Phe	148 (*143*)	5.0	12.2	19.1	11.3
Asp–Tyr–Phe	146 (*174*)	5.0	12.0	15.5	11.1
Asp–His–His	117 (*49*) ***	4.0	9.6	16.7	23.5
Glu–Tyr–Tyr	112 (*125*)	3.8	6.8	11.9	16.2
Asp–His–Phe	111 (*128*)	3.8	9.1	15.8	8.4
Asp–Trp–Phe	110 (*97*)	3.7	9.0	21.0	8.4
Glu–His–Phe	104 (*175*) ***	3.5	6.3	14.8	7.9
Glu–Trp–His	92 (*50*) ***	3.1	5.5	17.5	18.5
Glu–Trp–Trp	83 (*50*) **	2.8	5.0	15.8	18.8
Asp–Phe–Tyr	80 (*76*)	2.7	6.6	10.3	11.5
Asp–His–Trp	77 (*48*) *	2.6	6.3	11.0	17.5
Glu–Trp–Phe	76 (*132*) ***	2.6	4.6	14.5	5.8
Glu–His–Tyr	76 (*92*)	2.6	4.6	10.8	11.0
Asp–Tyr–Trp	73 (*66*)	2.5	6.0	7.7	16.6
Asp–His–Tyr	69 (*68*)	2.3	5.7	9.8	10.0

Asp–Tyr–Tyr	67 (*92*)	2.3	5.5	7.1	9.7
Glu–His–Trp	63 (*66*)	2.1	3.8	9.0	14.3
Glu–His–His	62 (*67*)	2.1	3.7	8.8	12.5
Glu–Phe–His	59 (*74*)	2.0	3.6	7.6	11.9
Glu–Trp–Tyr	49 (*70*)	1.7	3.0	9.3	7.1
Asp–Phe–His	43 (*54*)	1.5	3.5	5.5	8.7
Glu–Tyr–His	42 (*90*) ***	1.4	2.5	4.4	8.5
Asp–Trp–His	38 (*37*)	1.3	3.1	7.2	7.6
Asp–Tyr–His	37 (*66*) **	1.3	3.0	3.9	7.4
Asp–Trp–Trp	36 (*37*)	1.2	3.0	6.9	8.2
Glu–Tyr–Trp	34 (*89*) ***	1.2	2.1	3.6	7.7
Asp–Phe–Trp	34 (*54*) *	1.2	2.8	4.4	7.7
Glu–Phe–Trp	33 (*74*) ***	1.1	2.0	4.3	7.5
Asp–Trp–Tyr	30 (*51*) *	1.0	2.5	5.7	4.3
SO_4–His–Phe	8 (*3*)	0.3	26.7	1.1	0.6
Cl–Tyr–Phe	8 (*3*)	0.3	34.8	0.8	0.6
PO_4–His–Phe	6 (*2*)	0.2	60.0	0.9	0.5
SO_4–Tyr–Phe	6 (*4*)	0.2	20.0	0.6	0.5
Cl–Phe–Phe	3 (*3*)	0.1	13.0	0.4	0.2
NO_3–Tyr–Phe	3 (*1*)	0.1	42.9	0.3	0.2
Cl–Phe–His	3 (*1*)	0.1	13.0	0.4	0.6
SO_4–His–Tyr	3 (*2*)	0.1	10.0	0.4	0.4
SO_4–Tyr–Tyr	3 (*2*)	0.1	10.0	0.3	0.4
SO_4–Trp–Trp	3 (*1*)	0.1	10.0	0.6	0.7
Cl–Trp–Tyr	3 (*1*)	0.1	13.0	0.6	0.4
SO_4–Phe–His	2 (*1*)	0.1	6.7	0.3	0.4
SO_4–Phe–Phe	2 (*4*)	0.1	6.7	0.3	0.2
NO_3–Tyr–Tyr	2 (*1*)	0.1	28.6	0.2	0.3
SO_4–His–Trp	1 (*1*)	0.0	3.3	0.1	0.2
PO_4–Trp–Phe	1 (*1*)	0.0	10.0	0.2	0.1
PO_4–His–His	1 (*1*)	0.0	10.0	0.1	0.2

NO_3–Phe–Trp	1 (*0*)	0.0	14.3	0.1	0.2
Cl–Tyr–Tyr	1 (*2*)	0.0	4.3	0.1	0.1
Cl–His–His	1 (*1*)	0.0	4.3	0.1	0.2
Cl–His–Phe	1 (*2*)	0.0	4.3	0.1	0.1
CO_3–His–Trp	1 (*0*)	0.0	100.0	0.1	0.2
SO_4–Trp–Phe	1 (*2*)	0.0	3.3	0.2	0.1
Cl–Trp–Phe	1 (*2*)	0.0	4.3	0.2	0.1
SO_4–Phe–Trp	1 (*1*)	0.0	3.3	0.1	0.2
Cl–Phe–Tyr	1 (*1*)	0.0	4.3	0.1	0.1
Cl–Phe–Trp	1 (*1*)	0.0	4.3	0.1	0.2
PO_4–Trp–Tyr	1 (*1*)	0.0	10.0	0.2	0.1
NO_3–Trp–Tyr	1 (*0*)	0.0	14.3	0.2	0.1
PO_4–Tyr–Tyr	1 (*1*)	0.0	10.0	0.1	0.1

Supplementary Table 23. Interacting residues from the search of anion–π–π surface interactions in proteins, their amounts (central+terminal), and their respective percentages for the anions, central, and terminal aromatic moieties.

**Interacting residues**	**Amount**	**%A^-**	**%π_c**	**%π_t**
Phe	287 (97+190)	-	27.4	53.7
Glu	205	57.9	-	-
Tyr	180 (109+71)	-	30.8	20.0
Asp	149	42.1	-	-
His	142 (83+59)	-	23.4	16.7
Trp	99 (65+34)	-	18.4	9.6

Supplementary Table 24. Ternary inter-chain anion–π–π interactions in proteins. Triads of interacting residues and their occurrences in number (amount), percentage (%), and residues' representativities for each distinct anion (%A^-), central (%π_c), and terminal (%π_t) π-systems. The expected amount of each interaction pair, according to its relative abundance, and the statistical significance are shown (Methods).

Interaction	Amount (*expected*)	%	%A^-	%π_c	%π_t
Glu–Tyr–Phe	46 (*34*)	13.0	22.4	42.2	24.2
Asp–Trp–Phe	41 (*15*) ***	11.6	27.5	63.1	21.6
Glu–Phe–Phe	38 (*30*)	10.7	18.5	39.2	20.0
Glu–His–His	23 (*8*) **	6.5	11.2	27.7	39.0
Asp–Phe–Phe	22 (*22*)	6.2	14.8	22.7	11.6
Asp–Tyr–Phe	21 (*25*)	5.9	14.1	19.3	11.1
Glu–Phe–Tyr	20 (*11*)	5.6	9.8	20.6	28.2
Glu–Tyr–Tyr	14 (*13*)	4.0	6.8	12.8	19.7
Glu–His–Tyr	12 (*10*)	3.4	5.9	14.5	16.9
Asp–His–Trp	10 (*3*)	2.8	6.7	12.0	29.4
Asp–Tyr–Tyr	9 (*9*)	2.5	6.0	8.3	12.7
Glu–Tyr–His	9 (*11*)	2.5	4.4	8.3	15.3
Asp–His–His	9 (*6*)	2.5	6.0	10.8	15.3
Glu–His–Phe	8 (*26*) **	2.3	3.9	9.6	4.2
Asp–His–Phe	8 (*19*) *	2.3	5.4	9.6	4.2
Glu–Trp–Trp	8 (*4*)	2.3	3.9	12.3	23.5
Glu–His–Trp	7 (*5*)	2.0	3.4	8.4	20.6
Glu–Phe–His	7 (*9*)	2.0	3.4	7.2	11.9
Asp–His–Tyr	6 (*7*)	1.7	4.0	7.2	8.5
Glu–Trp–Phe	6 (*20*) **	1.7	2.9	9.2	3.2
Asp–Phe–His	5 (*7*)	1.4	3.4	5.2	8.5
Asp–Phe–Tyr	5 (*8*)	1.4	3.4	5.2	7.0
Glu–Tyr–Trp	4 (*6*)	1.1	2.0	3.7	11.0

Asp–Tyr–Trp	3 (*4*)	0.8	2.0	2.8	8.8
Asp–Trp–Tyr	3 (*5*)	0.8	2.0	4.6	4.2
Asp–Tyr–His	3 (*8*)	0.8	2.0	2.8	5.1
Asp–Trp–Trp	2 (*3*)	0.6	1.3	3.1	5.9
Asp–Trp–His	2 (*5*)	0.6	1.3	3.1	3.4
Glu–Trp–Tyr	2 (*8*)	0.6	1.0	3.1	2.8
Glu–Trp–His	1 (*6*)	0.3	0.5	1.5	1.7
